# Control chart for half normal and half exponential power distributed process

**DOI:** 10.1038/s41598-023-35884-0

**Published:** 2023-05-27

**Authors:** Muhammad Naveed, Muhammad Azam, Nasrullah Khan, Muhammad Aslam, Muhammad Saleem, Muhammad Saeed

**Affiliations:** 1 Department of Statistics, National College of Business Administration and Economics, Lahore, 54660 Pakistan; 2 Department of Statistics, Govt Graduate College (B) Gulberg, Lahore, Pakistan; 3 Department of Statistics and Computer Science, University of Veterinary and Animal Sciences, Lahore, 54000 Pakistan; 4 College of Statistical Science, University of the Punjab, Lahore, Pakistan; 5 Department of Statistics, Faculty of Science, King Abdulaziz University, Jeddah, 21551 Saudi Arabia; 6 Department of Industrial Engineering, Faculty of Engineering-Rabigh, King Abdulaziz University, Jeddah, 21589 Saudi Arabia; 7 Department of Statistics, Minhaj University, Lahore, 54770 Pakistan

**Keywords:** Engineering, Mathematics and computing

## Abstract

In this manuscript, we construct an attribute control chart (ACC) for the number of defective items using time-truncated life tests (TTLT) when the lifetime of a manufacturing item follows two lifetime data distributions: the half-normal distribution (HND) and the half-exponential power distribution (HEPD). To assess the potential of the proposed charts, necessary derivations are made to obtain the value of the average run length (ARL) when the production process is in-control and out-of-control. The performance of the presented charts is evaluated for different sample sizes, control coefficients, and truncated constants for shifted phases in terms of ARL. The behavior of ARLs is studied for the shifted process by introducing shifts in its parameters. The advantages of the proposed HEPD-based chart are discussed in the form of ARLs with HND and Exponential Distribution (ED) based ACCs under TTLT, showing the excellent assessment of the proposed chart. Additionally, the advantages of another proposed ACC using HND are compared with ED-based ACC, and the findings support the HND in the form of smaller ARLs. Finally, simulation testing and real-life implementation are also discussed for functional purposes.

## Introduction

A control chart (CC) is a graph that portrays whether an ongoing production process meets the intended specifications, and if not, the degree by which it deviates from those specifications. A CC is considered more effective if it can identify variations more rapidly. CCs are mainly divided into two types based on the nature of the dataset. If the quality nature of the study variable is in a quantifiable structure, such as the weight of an item or the height of a plastic jug, then variable CCs are used to inspect the ongoing process. Whereas, when we face a situation where we classify the manufacturing unit as either good or damaged, ACCs are preferred. The advantage of using ACC over variable CC is that it quickly analyzes the outcomes by reducing cost and time since it only requires defining units as good or defective. Many researchers have extensively studied the use of ACCs, for instance Ref. ^1^ designed the np control chart with curtailment to enhance its effectiveness while keeping false alarm rates at a specified level. The effectiveness of the proposed charting structure is measured by calculating the OOC average time to signal (ATS) in steady-state mode. Later on Ref. ^2^ proposed another np chart using multiple dependent state (MDS) sampling. This CC involves two pairs of control limits whose parameters are established based on the desired IC ARL. Thereafter Ref. ^3^ introduced another ACC based on multiple-dependent state repetitive sampling (MDSRS). This control chart is more capable of detecting smaller process shifts than existing ACCs. Later on Ref. ^4^ developed a variable batch-size ACC to monitor the non-conforming items. The CC coefficient was determined through non-linear optimization and used to calculate the ARL. Subsequently Ref. ^5^ discussed the ACC using the neutrosophic statistical interval method. Later on, a comparison between the proposed chart and an existing chart was conducted in terms of neutrosophic average run length (NARL). Following that, a neutrosophic exponentially weighted moving average (NEWMA) CC for attribute data was introduced by Ref. ^6^. They employed neutrosophic Monte Carlo simulation to determine the NARL. Another advancement in ACC was presented by Ref. ^7^. The authors proposed ACC utilizing neutrosophic statistics to monitor blood components. The practical applications of the presented chart demonstrated that the proposed chart was effective, suitable, adaptable, and informative for monitoring blood components in uncertain environments.

In the assembling industry, testing the lifetime of finished products can be time-consuming, as quality personnel often have to wait until the testing time is complete. To address this issue, researchers have utilized the TTLT method to test the lifetime of manufacturing items using predefined time intervals. Literature review reveals that many researchers have developed CCs for attribute nature using TTLT under the assumption that the quality of interest follows some life data distributions as Ref. ^8^ developed an ACC for the Weibull distribution (WD) using the concept of TTLT and calculated the ARLs to assess the capability of the proposed idea. A real-life example was also incorporated in this context. After that Ref. ^9^ proposed the $np$ chart using life time data distribution named as Exponentiated Weibull Distribution (EWD) under TTLT. The effectiveness of the presented chart was compared to other existing CCs in the form of ARLs and found the better results. Again Ref. ^10^ applied TTLT to the Pareto distribution of the second kind to develop the np chart. The researchers assumed that the lifetime of the product followed this distribution with a known or unknown shape parameter. The researchers conducted a simulation study to demonstrate how well the proposed control chart could monitor non-conforming items in industries. Later on TTLT technique has been used by Ref. ^11^. The author applied the TTLT to Exponentiated half logistic distribution for the construction of ACC. The performance of the proposed charting structure was assessed using a Monte Carlo simulation to determine its ARL. Additionally, the evaluation of the proposed CC was also discussed using simulated data sets for industrial purposes. More work using the concept of TTLT can be seen in Ref. ^12^. The researchers proposed ACC using neutrosophic Weibull distribution to monitor the process more efficiently. The authors presented an example related to automobile manufacturing to demonstrate the applicability of the proposal. Recently Ref.^13^ presented an attribute np control chart (CC) in their research, which was based on multiple dependent state repetitive sampling (MDSRS) for the purpose of monitoring the lifetime of products using TTLT. The study assumed that the lifetime of the product could be modeled using three different lifetime distributions: Weibull, gamma, and Pareto distributions of the second kind with known shape parameters. The performance of the chart was evaluated using the OOC average run length and compared to the results of the CC designed under a single sampling procedure, which showed outstanding performance of the proposed idea.

The existing work on the control charts using TTLT has limitations in that these charts cannot be applied when the lifetime does not follows the half-normal distribution and the half-exponential power distribution. Notably, these distributions have not been explored in the existing literature concerning the development of CCs under TTLT. Motivated by previous research that explores the use of ACCs through TTLT in manufacturing industries using various life data distributions, in this paper, we will develop ACCs using two widely recognized life data distributions: the half-normal distribution and the half-exponential power distribution. We will discuss the application of the proposed control charts using real data.

The rest of the paper is organized as follows: “Introduction of two life data distribution” section, provides a brief overview of the two life data distributions. “Designing of proposed control charts” section, describes the proposed CC design under the assumption that its parameters are shifted. “Results discussion” section, discusses the advantages of the presented chart. “Advantages of proposed control charts” section, presents the results of a simulation study conducted to evaluate the effectiveness of the suggested CC. “Simulation study” section, presents a real-world application of the recommended chart. Finally, concluding remarks are discussed in “Real life examples” section.

## Introduction of two life data distribution

In this section, we present a concise introduction based on HND and HEPD. In “Half normal distribution (HND)” section, we discuss the application of HND in the field of statistical quality control, and in “Half exponential power distribution (HEPD)” section, we discuss the application of HEPD in the field of SQC.

### Half normal distribution (HND)

Half Normal Distribution (HND) is broadly used distribution for modeling life data and was developed by Ref.^14^. They studied its properties and application in quality control. it is also used when we are handling the data under fatigue ^15^. Fatigue is the structural destruction that happens when a material is in constant exposure to stress. The HND is a special case of normal distribution. The probability distribution of the half normally distributed variable $t$ is given by

1 $$f\left( {t,\alpha } \right) = \frac{1}{\alpha }\sqrt {\frac{2}{\pi }} exp\left( {\frac{{ - t^{2} }}{{2\alpha^{2} }}} \right) ,\quad t \ge 0, \alpha > 0$$

here $\alpha$ is the scale parameter. The CDF denoted by $F\left(t\right)$ is given by

2 $$F\left( t \right) = erf\left( {\frac{t}{\alpha \sqrt 2 }} \right)$$

The $erf$ is error function defined as

3 $$erf\left( x \right) = \frac{2}{\sqrt \pi }\int_{0}^{x} {{\text{exp}}( - t^{2} ) dt}$$

The mean and variance of HND are given by

4 $$\mu = E\left( t \right) = \alpha \sqrt {\frac{2}{\pi }}$$

5 $$var\left( t \right) = \left( {\frac{\pi - 2}{\pi }} \right)\alpha^{2}$$

The application of HND can also be seen in the area of sports science, fiber buckling, physiology, blowfly, stochastic frontier model and particularly in the area of reliability. The application of HND in the area of ASPs can be seen in Refs. ^16–19^.

### Half exponential power distribution (HEPD)

The HEPD is the positive truncation of Exponential Power Distribution developed by Ref. ^20^. This is the generalization of HND and ED for non-negative variables. HEPD is extensively used in the field of reliability and quality control like Ref. ^21^ developed the sampling plan for HEPD using TTLT. The author discussed its operating characteristic function and associated risks. Later on, real life example has also been discussed for practical implementation. After that Ref. ^22^ proposed double acceptance sampling plan for HEPD using TTLT. The probability distribution function (pdf) and cumulative distribution function (cdf) of HEPD are given by

6 $$f\left( t \right) = \frac{{\lambda^{{1 - \frac{1}{\lambda }}} }}{{\upalpha \Gamma \left( {\frac{1}{\lambda }} \right)}}e^{{ - \frac{{t^{\lambda } }}{{\lambda \alpha^{\lambda } }}}} ;\quad t \ge 0, \alpha > 0, \lambda > 0$$

7 $$F\left( t \right) = \frac{{\gamma \left( {\frac{1 }{\lambda }, \frac{{t^{\lambda } }}{{\lambda \alpha^{\lambda } }}} \right)}}{{{\Gamma }\left( {{\raise0.7ex\hbox{$1$} \!\mathord{\left/ {\vphantom {1 \lambda }}\right.\kern-0pt} \!\lower0.7ex\hbox{$\lambda $}}} \right)}}$$

here $\alpha$ is the scale parameter and $\lambda$ is the shape parameter. HEPD is converted to the ED when the value of its shape parameter $\lambda =1$ and transformed to HND for $\lambda =2$. The mean of HEPD is as follows

8 $$\mu = E\left( t \right) = \frac{{\alpha \lambda^{{{\raise0.7ex\hbox{$1$} \!\mathord{\left/ {\vphantom {1 \lambda }}\right.\kern-0pt} \!\lower0.7ex\hbox{$\lambda $}}}} }}{{{\Gamma }\left( {{\raise0.7ex\hbox{$1$} \!\mathord{\left/ {\vphantom {1 \lambda }}\right.\kern-0pt} \!\lower0.7ex\hbox{$\lambda $}}} \right)}}{\Gamma }\left( {{\raise0.7ex\hbox{$2$} \!\mathord{\left/ {\vphantom {2 \lambda }}\right.\kern-0pt} \!\lower0.7ex\hbox{$\lambda $}}} \right)$$

Let the average life time of the failure products for the in-control (IC) process is ${\mu }_{0}$.

## Designing of proposed control charts

In this section, we propose two types of CCs based on HND and HEPD. Firstly, we describe the procedure for constructing the CC based on HND, followed by the procedure for constructing the CC based on HEPD.

### Designing of the control chart using Half Normal Distribution

Here, we suppose that the failure time of the manufacturing item follows the HND. The probability that an item fails before time ${t}_{0}$ is given by

9 $$P\left( {t < t_{0} } \right) = erf\left( {\frac{t}{\alpha \sqrt 2 }} \right){ }$$

replace the value of ${t}_{0}=h{\mu }_{0}$ where $h$ indicates the truncated constant for HND and the value of $\alpha$ in term of $\mu$ using Eq. (4), then Eq. (9) can be written as

10 $$\begin{aligned} & P\left( {t < t_{0} } \right) = erf\left( {\frac{{h\mu_{0} }}{{\frac{\mu \sqrt \pi }{{\sqrt 2 }}\sqrt 2 }}} \right) \\ & P\left( {t < t_{0} } \right) = erf\left( {\frac{h}{\sqrt \pi }\frac{{\mu_{0} }}{\mu }} \right) \\ \end{aligned}$$

The process is considered to be IC when $\mu ={\mu }_{0}$ then Eq. (10) becomes

11 $$P_{0HND} = P\left( {t < t_{0} } \right) = erf\left( {\frac{h}{\sqrt \pi }} \right)$$

The lower and upper control limits for the proposed $np$ charting structure using HND are as follows

12 $$LCL = \max \left[ {nP_{0HND} - k\sqrt {nP_{0HND} \left( {1 - P_{0HND} } \right)} } \right]$$

13 $$UCL = nP_{0HND} + k\sqrt {nP_{0HND} \left( {1 - P_{0HND} } \right)}$$

The working procedure for the presented chart is as follows

- *Stage 1* Draw an arbitrary sample from the process of size $$n$$ in each subgroup. Count the number of items (denoted by $$D)$$ that failed before reaching a predefined time $${t}_{0}=h{\mu }_{0}$$,
- *Stage 2* we declare the process as IC if $LCL\le D\le UCL$. Conversely, the process is declared as OOC if $D<LCL or D>UCL.$

It should be noted that the number of failure items $D$ for the IC process follows the Binomial Distribution (BD) with parameters $n and {P}_{0HND}$. Where ${P}_{0HND}$ is the probability that an item fails before time ${t}_{0}$ and $k$ is the control coefficient. In most cases, the value of ${P}_{0HND}$ is not known, so to tackle that situation we draw a preliminary sample from the IC process to determine the value of ${P}_{0HND}.$ Consequently the control limits which are designed for the practical objectives are

14 $$UCL = \overline{D} + k \sqrt {\overline{D}\left( {1 - \overline{D}/n} \right) }$$

15 $$LCL = max\left[ {0,\overline{D} - k \sqrt {\overline{D}\left( {1 - \overline{D}/n} \right) } } \right]$$

where $\overline{D }=\frac{\sum D}{n}$ denotes the mean failure time of items before time ${t}_{0}$ in a subgroup over a preliminary sample.

The probability that the manufacturing operation is declared to be IC when it is actually IC is given as

16 $$P_{in}^{0} = P\left( {LCL \le D \le UCL{|}P_{0HND} } \right)$$

17 $$P_{in}^{0} = \sum\nolimits_{d = LCL + 1}^{UCL} {\left( {\begin{array}{*{20}c} n \\ d \\ \end{array} } \right)\left( {erf\left( {\frac{h}{\sqrt \pi }} \right)} \right)^{d} \left( {1 - erf\left( {\frac{h}{\sqrt \pi }} \right)} \right)^{n - d} }$$

The competency of the proposed idea is assessed by using ARL. ARL for the IC process is given as

18 $${ARL}_{0HND}=\frac{1}{1-{P}_{in}^{0}}$$

#### Evaluation of the proposed HND based CC when its scale parameter is shifted

Here, we assume that the scale parameter of HND is shifted due to some extraneous source of variation from ${\alpha }_{0} to{\alpha }_{1}=c{\alpha }_{0}.$ Where $c$ is the amount of shift introduced. The probability that an item fails before reaching the specified time ${t}_{0}$ is represented by ${P}_{1HND}$ derived as,

Rewrite Eq. (10)

$$P\left(t<{t}_{0}\right)=erf\left(\frac{h}{\sqrt{\pi } }\frac{{\mu }_{0}}{\mu }\right)$$

Since, the scale parameter of HND is changed as ${\alpha }_{1}=c{\alpha }_{0}$, accordingly the mean level of HND is also shifted as ${\mu =\mu }_{1}$ where

19 $${\mu }_{1}={\alpha }_{1}\sqrt{\frac{2}{\pi }}$$

So, the above equation becomes

$$P\left(t<{t}_{0}\right)=erf\left(\frac{h}{\sqrt{\pi } }\frac{{\mu }_{0}}{{\mu }_{1}}\right)$$

substituting the value of ${\mu }_{1}={\alpha }_{1}\sqrt{\frac{2}{\pi }}$ and ${\mu }_{0}={\alpha }_{0}\sqrt{\frac{2}{\pi }}$, we get

20 $$\begin{aligned} & P\left( {t < t_{0} } \right) = erf\left( {\frac{h}{\sqrt \pi }\frac{{\alpha_{0} \sqrt {\frac{2}{\pi }} }}{{\alpha_{1} \sqrt {\frac{2}{\pi }} }}} \right){ } \\ & P\left( {t < t_{0} } \right) = erf\left( {\frac{h}{\sqrt \pi }\frac{{\alpha_{0} }}{{c\alpha_{0} }}} \right){ } \\ & P_{1HND} = P\left( {t < t_{0} } \right) = erf\left( {\frac{h}{c\sqrt \pi }} \right){ } \\ \end{aligned}$$

Now the probability that the process is IC when in fact it is switched due to the change in its scale parameter is calculated as

21 $$P_{in}^{1} = P\left( {LCL \le D \le UCL|p_{1HND} } \right)$$

22 $$P_{in}^{1} = \sum\nolimits_{d = LCL + 1}^{UCL} {\left( {\begin{array}{*{20}c} n \\ d \\ \end{array} } \right)\left( {erf\left( {\frac{h}{c\sqrt \pi }} \right)} \right)^{d} \left( {1 - erf\left( {\frac{h}{c\sqrt \pi }} \right)} \right)^{n - d} }$$

ARL for shifted process denoted ${ARL}_{1HND}$ is given as

23 $$ARL_{1HND} = \frac{1}{{1 - P_{in}^{1} }}$$

We currently employ the following algorithm to calculate the ARLs for suggested CC.

1. Fix the value of ARL $say ({r}_{0}=300, 370 etc)$
2. Determined the value of $h,k$ according to the given sample size $n$ for which ${ARL}_{0HND}$ in Eq. (18) is nearer to ${r}_{0}.$
3. Utilize the values of $h, c and n$ obtained in step 2 to calculate the value of ${ARL}_{1HND}$ using Eq. (23) for different values of c.

### Designing of the control chart using Half Exponential Power Distribution

Now, we assume that the failure time of the item follows the HEPD. The probability that an item fails before time ${t}_{0}$ is given by where ${t}_{0}={h}_{1}{\mu }_{0}$

24 $$P\left( {t < t_{0} } \right) = \frac{{\gamma \left( {{\raise0.7ex\hbox{${1 }$} \!\mathord{\left/ {\vphantom {{1 } \lambda }}\right.\kern-0pt} \!\lower0.7ex\hbox{$\lambda $}}, {\raise0.7ex\hbox{${t_{0}^{\lambda } }$} \!\mathord{\left/ {\vphantom {{t_{0}^{\lambda } } {\lambda \alpha^{\lambda } }}}\right.\kern-0pt} \!\lower0.7ex\hbox{${\lambda \alpha^{\lambda } }$}}} \right)}}{{{\Gamma }\left( {{\raise0.7ex\hbox{$1$} \!\mathord{\left/ {\vphantom {1 \lambda }}\right.\kern-0pt} \!\lower0.7ex\hbox{$\lambda $}}} \right)}}$$

Substituting the value of ${t}_{0}={h}_{1}{\mu }_{0}$ where ${h}_{1}$ represents the truncated constant for HEPD, also put the value of $\alpha$ in terms of $\mu$ using Eq. (8), then Eq. (24) can be written as

25 $$\begin{aligned} & P\left( {t < t_{0} } \right) = \frac{{\gamma \left( {{\raise0.7ex\hbox{$1$} \!\mathord{\left/ {\vphantom {1 {\lambda }}}\right.\kern-0pt} \!\lower0.7ex\hbox{${\lambda }$}},\frac{{\left( {h_{1} u_{0} } \right)^{\lambda } }}{{\lambda \left( {\frac{{\mu {\Gamma }\left( {{\raise0.7ex\hbox{$1$} \!\mathord{\left/ {\vphantom {1 \lambda }}\right.\kern-0pt} \!\lower0.7ex\hbox{$\lambda $}}} \right)}}{{{\Gamma }\left( {{\raise0.7ex\hbox{$2$} \!\mathord{\left/ {\vphantom {2 \lambda }}\right.\kern-0pt} \!\lower0.7ex\hbox{$\lambda $}}} \right)\lambda^{{{\raise0.7ex\hbox{$1$} \!\mathord{\left/ {\vphantom {1 \lambda }}\right.\kern-0pt} \!\lower0.7ex\hbox{$\lambda $}}}} }}} \right)^{\lambda } }}} \right)}}{{{\Gamma }\left( {{\raise0.7ex\hbox{$1$} \!\mathord{\left/ {\vphantom {1 \eta }}\right.\kern-0pt} \!\lower0.7ex\hbox{$\eta $}}} \right)}} \\ & P\left( {t < t_{0} } \right) = \frac{{\gamma \left( {{\raise0.7ex\hbox{$1$} \!\mathord{\left/ {\vphantom {1 {\lambda }}}\right.\kern-0pt} \!\lower0.7ex\hbox{${\lambda }$}},\frac{{h_{1}^{\lambda } {\Gamma }\left( {{\raise0.7ex\hbox{$2$} \!\mathord{\left/ {\vphantom {2 \lambda }}\right.\kern-0pt} \!\lower0.7ex\hbox{$\lambda $}}} \right)^{{\uplambda }} }}{{\left( {{\Gamma }\left( {{\raise0.7ex\hbox{$1$} \!\mathord{\left/ {\vphantom {1 \lambda }}\right.\kern-0pt} \!\lower0.7ex\hbox{$\lambda $}}} \right)} \right)^{{\uplambda }} }}\left( {\frac{{\mu_{0} }}{\mu }} \right)^{\lambda } } \right)}}{{{\Gamma }\left( {{\raise0.7ex\hbox{$1$} \!\mathord{\left/ {\vphantom {1 \lambda }}\right.\kern-0pt} \!\lower0.7ex\hbox{$\lambda $}}} \right)}} \\ \end{aligned}$$

The process is declared to be IC when $\mu ={\mu }_{0}$ (or $\lambda ={\lambda }_{0}$ and $\alpha ={\alpha }_{0}$) then Eq. (25) can be written as

26 $$P_{0HEPD} = P_{0} \left( {t < t_{0} } \right) = \frac{{\gamma \left( {{\raise0.7ex\hbox{$1$} \!\mathord{\left/ {\vphantom {1 {\lambda_{0} }}}\right.\kern-0pt} \!\lower0.7ex\hbox{${\lambda_{0} }$}},\frac{{h_{1}^{{\lambda_{0} }} {\Gamma }\left( {{\raise0.7ex\hbox{$2$} \!\mathord{\left/ {\vphantom {2 {\lambda_{0} }}}\right.\kern-0pt} \!\lower0.7ex\hbox{${\lambda_{0} }$}}} \right)^{{\lambda_{0} }} }}{{\left( {{\Gamma }\left( {{\raise0.7ex\hbox{$1$} \!\mathord{\left/ {\vphantom {1 {\lambda_{0} }}}\right.\kern-0pt} \!\lower0.7ex\hbox{${\lambda_{0} }$}}} \right)} \right)^{{\lambda_{0} }} }}} \right)}}{{{\Gamma }\left( {{\raise0.7ex\hbox{$1$} \!\mathord{\left/ {\vphantom {1 {\lambda_{0} }}}\right.\kern-0pt} \!\lower0.7ex\hbox{${\lambda_{0} }$}}} \right)}}$$

The lower and upper control limits for the presented $np$ chart using HEPD is as follows

27 $$LCL = \max \left[ {nP_{0HEPD} - k_{1} \sqrt {nP_{0HEPD} \left( {1 - P_{0HEPD} } \right)} } \right]$$

28 $$UCL = nP_{0HEPD} + k_{1} \sqrt {nP_{0HEPD} \left( {1 - P_{0HEPD} } \right)}$$

The execution for recommended charting structure is as follows:

- *Step 1* Choose a random sample of size $n$ from the process in each subgroup. Count the number of items (say ${D}_{1}$) that are rejected before reaching a preset time ${t}_{0}={h}_{1}{\mu }_{0}$
- *Step 2* Process is considered to be IC if $LCL\le {D}_{1}\le UCL$. Otherwise, the process is declared as OOC if ${D}_{1}<LCL or {D}_{1}>UCL.$

Here again ${D}_{1}$ the number of failure items follows the BD with parameters $n and {P}_{0HEPD}$. Where ${P}_{0HEPD}$ is the likelihood that an item fails before time ${t}_{0}$ and ${k}_{1}$ is the control coefficient. For the situation when the value of ${P}_{0HEPD}$ is unknown, we extract a preliminary sample from the IC process to compute the value of ${P}_{0HEPD}.$ As a result, the control limits which are used for realistic purposes are

29 $$UCL = \overline{D}_{1} + k_{1} \sqrt {\overline{D}_{1} \left( {1 - \overline{D}_{1} /n} \right) }$$

30 $$LCL = max\left[ {0,\overline{D}_{1} + k_{1} \sqrt {\overline{D}_{1} \left( {1 - \overline{D}_{1} /n} \right) } } \right]$$

where ${\overline{D} }_{1}=\frac{\sum {D}_{1}}{n}$ is the mean failure time of items in a subgroup before time ${t}_{0}$ over a preliminary sample. The probability that an item is considered to be IC when the process is working in normal conditions is given as

31 $$P_{in}^{0} = P\left( {LCL \le D_{1} \le UCL{|}P_{0HEPD} } \right)$$

32 $$P_{in}^{0} = \sum\nolimits_{{d_{1} = LCL + 1}}^{UCL} {\left( {\begin{array}{*{20}c} n \\ {d_{1} } \\ \end{array} } \right)} \left( {\frac{{\gamma \left( {{\raise0.7ex\hbox{$1$} \!\mathord{\left/ {\vphantom {1 {\lambda_{0} }}}\right.\kern-0pt} \!\lower0.7ex\hbox{${\lambda_{0} }$}},\frac{{h_{1}^{{\lambda_{0} }} {\Gamma }\left( {{\raise0.7ex\hbox{$2$} \!\mathord{\left/ {\vphantom {2 {\lambda_{0} }}}\right.\kern-0pt} \!\lower0.7ex\hbox{${\lambda_{0} }$}}} \right)^{{\lambda_{0} }} }}{{\left( {{\Gamma }\left( {{\raise0.7ex\hbox{$1$} \!\mathord{\left/ {\vphantom {1 {\lambda_{0} }}}\right.\kern-0pt} \!\lower0.7ex\hbox{${\lambda_{0} }$}}} \right)} \right)^{{\lambda_{0} }} }}} \right)}}{{{\Gamma }\left( {{\raise0.7ex\hbox{$1$} \!\mathord{\left/ {\vphantom {1 {\lambda_{0} }}}\right.\kern-0pt} \!\lower0.7ex\hbox{${\lambda_{0} }$}}} \right)}}} \right)^{d} \left( {1 - \frac{{\gamma \left( {{\raise0.7ex\hbox{$1$} \!\mathord{\left/ {\vphantom {1 {\lambda_{0} }}}\right.\kern-0pt} \!\lower0.7ex\hbox{${\lambda_{0} }$}},\frac{{h_{1}^{{\lambda_{0} }} {\Gamma }\left( {{\raise0.7ex\hbox{$2$} \!\mathord{\left/ {\vphantom {2 {\lambda_{0} }}}\right.\kern-0pt} \!\lower0.7ex\hbox{${\lambda_{0} }$}}} \right)^{{\lambda_{0} }} }}{{\left( {{\Gamma }\left( {{\raise0.7ex\hbox{$1$} \!\mathord{\left/ {\vphantom {1 {\lambda_{0} }}}\right.\kern-0pt} \!\lower0.7ex\hbox{${\lambda_{0} }$}}} \right)} \right)^{{\lambda_{0} }} }}} \right)}}{{{\Gamma }\left( {{\raise0.7ex\hbox{$1$} \!\mathord{\left/ {\vphantom {1 {\lambda_{0} }}}\right.\kern-0pt} \!\lower0.7ex\hbox{${\lambda_{0} }$}}} \right)}}} \right)^{n - d}$$

Consequently, ARL for the IC process denoted by ${ARL}_{0HEPD}$ is given as

33 $$ARL_{0HEPD} = \frac{1}{{1 - P_{in}^{0} }}$$

#### Evaluation of the suggested CC using HEPD when its scale parameter is changed

In this section, we assume that one of the parameters of HEPD, i.e., the scale parameter, has been changed from ${\alpha }_{0} to {\alpha }_{1}=q{\alpha }_{0}$ due to some variation, where $q$ denotes the amount of shift introduced. The probability of an item failing before the specified time ${t}_{0}$ is denoted by ${P}_{1HEPD}$, which can be calculated as follows:

Rewrite Eq. (25)

$$P\left( {t < t_{0} } \right) = \frac{{\gamma \left( {{\raise0.7ex\hbox{$1$} \!\mathord{\left/ {\vphantom {1 {\lambda }}}\right.\kern-0pt} \!\lower0.7ex\hbox{${\lambda }$}},\frac{{h_{1}^{\lambda } {\Gamma }\left( {{\raise0.7ex\hbox{$2$} \!\mathord{\left/ {\vphantom {2 \lambda }}\right.\kern-0pt} \!\lower0.7ex\hbox{$\lambda $}}} \right)^{{\uplambda }} }}{{\left( {{\Gamma }\left( {{\raise0.7ex\hbox{$1$} \!\mathord{\left/ {\vphantom {1 \lambda }}\right.\kern-0pt} \!\lower0.7ex\hbox{$\lambda $}}} \right)} \right)^{{\uplambda }} }}\left( {\frac{{\mu_{0} }}{\mu }} \right)^{\lambda } } \right)}}{{{\Gamma }\left( {{\raise0.7ex\hbox{$1$} \!\mathord{\left/ {\vphantom {1 \lambda }}\right.\kern-0pt} \!\lower0.7ex\hbox{$\lambda $}}} \right)}}$$

Since, the scale level is changed from ${\alpha }_{1}=q{\alpha }_{0}$, as a result the mean value of HEPD is also changed as ${{\mu }_{0}=\mu }_{1}$ where

34 $$\begin{aligned} & \mu_{1} = \frac{{\alpha_{1} \left( {\lambda_{0}^{{{\raise0.7ex\hbox{$1$} \!\mathord{\left/ {\vphantom {1 {\lambda_{0} }}}\right.\kern-0pt} \!\lower0.7ex\hbox{${\lambda_{0} }$}}}} } \right) }}{{{\Gamma }\left( {{\raise0.7ex\hbox{$1$} \!\mathord{\left/ {\vphantom {1 {\lambda_{0} }}}\right.\kern-0pt} \!\lower0.7ex\hbox{${\lambda_{0} }$}}} \right)}}{\Gamma }\left( {{\raise0.7ex\hbox{$2$} \!\mathord{\left/ {\vphantom {2 {\lambda_{0} }}}\right.\kern-0pt} \!\lower0.7ex\hbox{${\lambda_{0} }$}}} \right) \\ & P\left( {t < t_{0} } \right) = \frac{{\gamma \left( {{\raise0.7ex\hbox{$1$} \!\mathord{\left/ {\vphantom {1 {\lambda }}}\right.\kern-0pt} \!\lower0.7ex\hbox{${\lambda }$}},\frac{{h_{1}^{\lambda } {\Gamma }\left( {{\raise0.7ex\hbox{$2$} \!\mathord{\left/ {\vphantom {2 \lambda }}\right.\kern-0pt} \!\lower0.7ex\hbox{$\lambda $}}} \right)^{{\uplambda }} }}{{\left( {{\Gamma }\left( {{\raise0.7ex\hbox{$1$} \!\mathord{\left/ {\vphantom {1 \lambda }}\right.\kern-0pt} \!\lower0.7ex\hbox{$\lambda $}}} \right)} \right)^{{\uplambda }} }}\left( {\frac{{\mu_{0} }}{{\mu_{1} }}} \right)^{\lambda } } \right)}}{{{\Gamma }\left( {{\raise0.7ex\hbox{$1$} \!\mathord{\left/ {\vphantom {1 \lambda }}\right.\kern-0pt} \!\lower0.7ex\hbox{$\lambda $}}} \right)}} \\ \end{aligned}$$

Substituting the values $\mu_{1} , \lambda = \lambda_{0} \,{\text{and}}\, \alpha_{1} = q\alpha_{0}$ in the above equation we have

$$P\left( {t < t_{0} } \right) = \frac{{\gamma \left( {{\raise0.7ex\hbox{$1$} \!\mathord{\left/ {\vphantom {1 {\lambda_{0} }}}\right.\kern-0pt} \!\lower0.7ex\hbox{${\lambda_{0} }$}},\frac{{h_{1}^{{\lambda_{0} }} {\Gamma }\left( {{\raise0.7ex\hbox{$2$} \!\mathord{\left/ {\vphantom {2 {\lambda_{0} }}}\right.\kern-0pt} \!\lower0.7ex\hbox{${\lambda_{0} }$}}} \right)^{{\lambda_{0} }} }}{{\left( {{\Gamma }\left( {{\raise0.7ex\hbox{$1$} \!\mathord{\left/ {\vphantom {1 {\lambda_{0} }}}\right.\kern-0pt} \!\lower0.7ex\hbox{${\lambda_{0} }$}}} \right)} \right)^{{\lambda_{0} }} }}\left( {\frac{{\mu_{0} }}{{\frac{{q\alpha_{0} \left( {\lambda_{0}^{{1/\lambda_{0} }} } \right) }}{{{\Gamma }\left( {{\raise0.7ex\hbox{$1$} \!\mathord{\left/ {\vphantom {1 {\lambda_{0} }}}\right.\kern-0pt} \!\lower0.7ex\hbox{${\lambda_{0} }$}}} \right)}}{\Gamma }\left( {{\raise0.7ex\hbox{$2$} \!\mathord{\left/ {\vphantom {2 {\lambda_{0} }}}\right.\kern-0pt} \!\lower0.7ex\hbox{${\lambda_{0} }$}}} \right)}}} \right)^{{\lambda_{0} }} } \right)}}{{{\Gamma }\left( {{\raise0.7ex\hbox{$1$} \!\mathord{\left/ {\vphantom {1 {\lambda_{0} }}}\right.\kern-0pt} \!\lower0.7ex\hbox{${\lambda_{0} }$}}} \right)}}$$

Put $$\mu_{0} = \frac{{\alpha_{0} \lambda_{0}^{{1/\lambda_{0} }} }}{{\Gamma \left( {{\raise0.7ex\hbox{$1$} \!\mathord{\left/ {\vphantom {1 {\lambda_{0} }}}\right.\kern-0pt} \!\lower0.7ex\hbox{${\lambda_{0} }$}}} \right)}}\Gamma \left( {{\raise0.7ex\hbox{$2$} \!\mathord{\left/ {\vphantom {2 {\lambda_{0} }}}\right.\kern-0pt} \!\lower0.7ex\hbox{${\lambda_{0} }$}}} \right)$$ in the above equation

35 $$\begin{aligned} & P\left( {t < t_{0} } \right) = \frac{{\gamma \left( {{\raise0.7ex\hbox{$1$} \!\mathord{\left/ {\vphantom {1 {\lambda_{0} }}}\right.\kern-0pt} \!\lower0.7ex\hbox{${\lambda_{0} }$}},\frac{{h_{1}^{{\lambda_{0} }} {\Gamma }\left( {{\raise0.7ex\hbox{$2$} \!\mathord{\left/ {\vphantom {2 {\lambda_{0} }}}\right.\kern-0pt} \!\lower0.7ex\hbox{${\lambda_{0} }$}}} \right)^{{\lambda_{0} }} }}{{\left( {{\Gamma }\left( {{\raise0.7ex\hbox{$1$} \!\mathord{\left/ {\vphantom {1 {\lambda_{0} }}}\right.\kern-0pt} \!\lower0.7ex\hbox{${\lambda_{0} }$}}} \right)} \right)^{{\lambda_{0} }} }}\left( {\frac{{\frac{{\alpha_{0} \lambda_{0}^{{1/\lambda_{0} }} }}{{{\Gamma }\left( {{\raise0.7ex\hbox{$1$} \!\mathord{\left/ {\vphantom {1 {\lambda_{0} }}}\right.\kern-0pt} \!\lower0.7ex\hbox{${\lambda_{0} }$}}} \right)}}{\Gamma }\left( {{\raise0.7ex\hbox{$2$} \!\mathord{\left/ {\vphantom {2 {\lambda_{0} }}}\right.\kern-0pt} \!\lower0.7ex\hbox{${\lambda_{0} }$}}} \right){ }}}{{\frac{{q\alpha_{0} \left( {\lambda_{0}^{{1/\lambda_{0} }} } \right) }}{{{\Gamma }\left( {{\raise0.7ex\hbox{$1$} \!\mathord{\left/ {\vphantom {1 {\lambda_{0} }}}\right.\kern-0pt} \!\lower0.7ex\hbox{${\lambda_{0} }$}}} \right)}}{\Gamma }\left( {{\raise0.7ex\hbox{$2$} \!\mathord{\left/ {\vphantom {2 {\lambda_{0} }}}\right.\kern-0pt} \!\lower0.7ex\hbox{${\lambda_{0} }$}}} \right)}}} \right)^{{\lambda_{0} }} } \right)}}{{{\Gamma }\left( {{\raise0.7ex\hbox{$1$} \!\mathord{\left/ {\vphantom {1 \lambda }}\right.\kern-0pt} \!\lower0.7ex\hbox{$\lambda $}}} \right)}} \\ & P\left( {t < t_{0} } \right) = \frac{{\gamma \left( {{\raise0.7ex\hbox{$1$} \!\mathord{\left/ {\vphantom {1 {\lambda_{0} }}}\right.\kern-0pt} \!\lower0.7ex\hbox{${\lambda_{0} }$}},\frac{{h_{1}^{{\lambda_{0} }} {\Gamma }\left( {{\raise0.7ex\hbox{$2$} \!\mathord{\left/ {\vphantom {2 {\lambda_{0} }}}\right.\kern-0pt} \!\lower0.7ex\hbox{${\lambda_{0} }$}}} \right)^{{\lambda_{0} }} }}{{q^{{\lambda_{0} }} \left( {{\Gamma }\left( {{\raise0.7ex\hbox{$1$} \!\mathord{\left/ {\vphantom {1 {\lambda_{0} }}}\right.\kern-0pt} \!\lower0.7ex\hbox{${\lambda_{0} }$}}} \right)} \right)^{{\lambda_{0} }} }}} \right)}}{{{\Gamma }\left( {{\raise0.7ex\hbox{$1$} \!\mathord{\left/ {\vphantom {1 {\lambda_{0} }}}\right.\kern-0pt} \!\lower0.7ex\hbox{${\lambda_{0} }$}}} \right)}} \\ & P_{1HEPD} = P\left( {t < t_{0} } \right) = \frac{{\gamma \left( {{\raise0.7ex\hbox{$1$} \!\mathord{\left/ {\vphantom {1 {\lambda_{0} }}}\right.\kern-0pt} \!\lower0.7ex\hbox{${\lambda_{0} }$}},\frac{{h_{1}^{{\lambda_{0} }} {\Gamma }\left( {{\raise0.7ex\hbox{$2$} \!\mathord{\left/ {\vphantom {2 {\lambda_{0} }}}\right.\kern-0pt} \!\lower0.7ex\hbox{${\lambda_{0} }$}}} \right)^{{\lambda_{0} }} }}{{q^{{\lambda_{0} }} \left( {{\Gamma }\left( {{\raise0.7ex\hbox{$1$} \!\mathord{\left/ {\vphantom {1 {\lambda_{0} }}}\right.\kern-0pt} \!\lower0.7ex\hbox{${\lambda_{0} }$}}} \right)} \right)^{{\lambda_{0} }} }}} \right)}}{{{\Gamma }\left( {{\raise0.7ex\hbox{$1$} \!\mathord{\left/ {\vphantom {1 {\lambda_{0} }}}\right.\kern-0pt} \!\lower0.7ex\hbox{${\lambda_{0} }$}}} \right)}} \\ \end{aligned}$$

Now the probability that operation is IC for shifted process due to the change in scale parameter is as follows:

36 $$P_{in}^{1} = P\left( {LCL \le D_{1} \le UCL{|}p_{1HEPD} } \right)$$

37 $$= \mathop \sum \limits_{{d_{1} = LCL + 1}}^{UCL} \left( {\begin{array}{*{20}c} n \\ {d_{1} } \\ \end{array} } \right)\left( {\frac{{\gamma \left( {{\raise0.7ex\hbox{$1$} \!\mathord{\left/ {\vphantom {1 {\lambda_{0} }}}\right.\kern-0pt} \!\lower0.7ex\hbox{${\lambda_{0} }$}},\frac{{h_{1}^{{\lambda_{0} }} {\Gamma }\left( {{\raise0.7ex\hbox{$2$} \!\mathord{\left/ {\vphantom {2 {\lambda_{0} }}}\right.\kern-0pt} \!\lower0.7ex\hbox{${\lambda_{0} }$}}} \right)^{{\lambda_{0} }} }}{{q^{{\lambda_{0} }} \left( {{\Gamma }\left( {{\raise0.7ex\hbox{$1$} \!\mathord{\left/ {\vphantom {1 {\lambda_{0} }}}\right.\kern-0pt} \!\lower0.7ex\hbox{${\lambda_{0} }$}}} \right)} \right)^{{\lambda_{0} }} }}} \right)}}{{{\Gamma }\left( {{\raise0.7ex\hbox{$1$} \!\mathord{\left/ {\vphantom {1 {\lambda_{0} }}}\right.\kern-0pt} \!\lower0.7ex\hbox{${\lambda_{0} }$}}} \right)}}{ }} \right)^{{d_{1} }} \left( {1 - \frac{{\gamma \left( {{\raise0.7ex\hbox{$1$} \!\mathord{\left/ {\vphantom {1 {\lambda_{0} }}}\right.\kern-0pt} \!\lower0.7ex\hbox{${\lambda_{0} }$}},\frac{{h_{1}^{{\lambda_{0} }} {\Gamma }\left( {{\raise0.7ex\hbox{$2$} \!\mathord{\left/ {\vphantom {2 {\lambda_{0} }}}\right.\kern-0pt} \!\lower0.7ex\hbox{${\lambda_{0} }$}}} \right)^{{\lambda_{0} }} }}{{q^{{\lambda_{0} }} \left( {{\Gamma }\left( {{\raise0.7ex\hbox{$1$} \!\mathord{\left/ {\vphantom {1 {\lambda_{0} }}}\right.\kern-0pt} \!\lower0.7ex\hbox{${\lambda_{0} }$}}} \right)} \right)^{{\lambda_{0} }} }}} \right)}}{{{\Gamma }\left( {{\raise0.7ex\hbox{$1$} \!\mathord{\left/ {\vphantom {1 {\lambda_{0} }}}\right.\kern-0pt} \!\lower0.7ex\hbox{${\lambda_{0} }$}}} \right)}}{ }} \right)^{{n - d_{1} }}$$

The ARL for shifted process denoted by ${ARL}_{1HEPD}$ is given as follows

38 $$ARL_{1HEPD} = \frac{1}{{1 - P_{in}^{1} }}$$

Now, we utilize the subsequent algorithm to process the Tables of ARLs for suggested CC based on HEPD when its scale parameter is shifted.

1. Firstly, we set the value of the shape parameter ${\lambda }_{0}$ and ARL $say ({r}_{0}=300, 370 etc)$
2. After that we determine the values ${h}_{1},{k}_{1}$ according to the given sample size $n$ for which ${ARL}_{0HEPD}$ in Eq. (33) is close to ${r}_{0}.$
3. Use the values of ${h}_{1},{k}_{1}nd n$ acquired in step 2 to ascertain the value of ${ARL}_{1HEPD}$ using Eq. (38) for different values of $q$.

#### Evaluation of proposed CC when its shape parameter is transferred

In this case, we presume that the shape parameter (the second parameter) of HEPD has changed due to some deviation. Suppose it is changed from ${\lambda }_{0} to {\lambda }_{1}$. Where ${\lambda }_{1}=w{\lambda }_{0}.$ Now, the probability of an object collapsing sooner than the predefined period ${t}_{0}$ designated by ${P}_{2HEPD}$ is calculated as.

Rewrite Eq. (25)

$$P\left( {t < t_{0} } \right) = \frac{{\gamma \left( {{\raise0.7ex\hbox{$1$} \!\mathord{\left/ {\vphantom {1 {\lambda }}}\right.\kern-0pt} \!\lower0.7ex\hbox{${\lambda }$}},\frac{{h_{1}^{\lambda } \Gamma \left( {{\raise0.7ex\hbox{$2$} \!\mathord{\left/ {\vphantom {2 \lambda }}\right.\kern-0pt} \!\lower0.7ex\hbox{$\lambda $}}} \right)^{\lambda } }}{{\left( {\Gamma \left( {{\raise0.7ex\hbox{$1$} \!\mathord{\left/ {\vphantom {1 \lambda }}\right.\kern-0pt} \!\lower0.7ex\hbox{$\lambda $}}} \right)} \right)^{\lambda } }}\left( {\frac{{\mu_{0} }}{\mu }} \right)^{\lambda } } \right)}}{{\Gamma \left( {{\raise0.7ex\hbox{$1$} \!\mathord{\left/ {\vphantom {1 \lambda }}\right.\kern-0pt} \!\lower0.7ex\hbox{$\lambda $}}} \right)}}$$

As the shape parameter is shifted from ${\lambda }_{1}=w{\lambda }_{0}$, so its mean level is also changed as ${\mu =\mu }_{1}$ where

39 $$\begin{aligned} & \mu_{1} = \frac{{\alpha_{0} \lambda_{1}^{{{\raise0.7ex\hbox{$1$} \!\mathord{\left/ {\vphantom {1 {\lambda_{1} }}}\right.\kern-0pt} \!\lower0.7ex\hbox{${\lambda_{1} }$}}}} }}{{{\Gamma }\left( {{\raise0.7ex\hbox{$1$} \!\mathord{\left/ {\vphantom {1 {\lambda_{1} }}}\right.\kern-0pt} \!\lower0.7ex\hbox{${\lambda_{1} }$}}} \right)}}{\Gamma }\left( {{\raise0.7ex\hbox{$2$} \!\mathord{\left/ {\vphantom {2 {\lambda_{1} }}}\right.\kern-0pt} \!\lower0.7ex\hbox{${\lambda_{1} }$}}} \right) \\ & P\left( {t < t_{0} } \right) = \frac{{\gamma \left( {{\raise0.7ex\hbox{$1$} \!\mathord{\left/ {\vphantom {1 {\lambda_{1} }}}\right.\kern-0pt} \!\lower0.7ex\hbox{${\lambda_{1} }$}},\left( {\frac{{h_{1}^{{\lambda_{1} }} {\Gamma }\left( {{\raise0.7ex\hbox{$2$} \!\mathord{\left/ {\vphantom {2 {\lambda_{1} }}}\right.\kern-0pt} \!\lower0.7ex\hbox{${\lambda_{1} }$}}} \right)^{{\lambda_{1} }} }}{{\left( {{\Gamma }\left( {{\raise0.7ex\hbox{$1$} \!\mathord{\left/ {\vphantom {1 {\lambda_{1} }}}\right.\kern-0pt} \!\lower0.7ex\hbox{${\lambda_{1} }$}}} \right)} \right)^{{\lambda_{1} }} }}} \right)\left( {\frac{{\mu_{0} }}{{\mu_{1} }}} \right)^{{\lambda_{1} }} } \right)}}{{{\Gamma }\left( {{\raise0.7ex\hbox{$1$} \!\mathord{\left/ {\vphantom {1 {\lambda_{1} }}}\right.\kern-0pt} \!\lower0.7ex\hbox{${\lambda_{1} }$}}} \right)}} \\ \end{aligned}$$

Substituting the values $$\mu_{1} ,\alpha = \alpha_{0}$$ and $$\mu_{0} = \frac{{\alpha_{0} \lambda_{0}^{{1/\lambda_{0} }} }}{{\Gamma \left( {{\raise0.7ex\hbox{$1$} \!\mathord{\left/ {\vphantom {1 {\lambda_{0} }}}\right.\kern-0pt} \!\lower0.7ex\hbox{${\lambda_{0} }$}}} \right)}}\Gamma \left( {{\raise0.7ex\hbox{$2$} \!\mathord{\left/ {\vphantom {2 {\lambda_{0} }}}\right.\kern-0pt} \!\lower0.7ex\hbox{${\lambda_{0} }$}}} \right)$$ in above equation

40 $$\begin{aligned} & P\left( {t < t_{0} } \right) = \frac{{\gamma \left( {{\raise0.7ex\hbox{$1$} \!\mathord{\left/ {\vphantom {1 {\lambda_{1} }}}\right.\kern-0pt} \!\lower0.7ex\hbox{${\lambda_{1} }$}}\left( {,\frac{{h_{1}^{{\lambda_{1} }} {\Gamma }\left( {{\raise0.7ex\hbox{$2$} \!\mathord{\left/ {\vphantom {2 {\lambda_{1} }}}\right.\kern-0pt} \!\lower0.7ex\hbox{${\lambda_{1} }$}}} \right)^{{\lambda_{1} }} }}{{\left( {{\Gamma }\left( {{\raise0.7ex\hbox{$1$} \!\mathord{\left/ {\vphantom {1 {\lambda_{1} }}}\right.\kern-0pt} \!\lower0.7ex\hbox{${\lambda_{1} }$}}} \right)} \right)^{{\lambda_{1} }} }}} \right)\left( {\frac{{\frac{{\alpha_{0} \lambda_{0}^{{{\raise0.7ex\hbox{$1$} \!\mathord{\left/ {\vphantom {1 {\lambda_{0} }}}\right.\kern-0pt} \!\lower0.7ex\hbox{${\lambda_{0} }$}}}} }}{{{\Gamma }\left( {{\raise0.7ex\hbox{$1$} \!\mathord{\left/ {\vphantom {1 {\lambda_{0} }}}\right.\kern-0pt} \!\lower0.7ex\hbox{${\lambda_{0} }$}}} \right)}}{\Gamma }\left( {{\raise0.7ex\hbox{$2$} \!\mathord{\left/ {\vphantom {2 {\lambda_{0} }}}\right.\kern-0pt} \!\lower0.7ex\hbox{${\lambda_{0} }$}}} \right)}}{{\frac{{\alpha_{0} \lambda_{1}^{{{\raise0.7ex\hbox{$1$} \!\mathord{\left/ {\vphantom {1 {\lambda_{1} }}}\right.\kern-0pt} \!\lower0.7ex\hbox{${\lambda_{1} }$}}}} }}{{{\Gamma }\left( {{\raise0.7ex\hbox{$1$} \!\mathord{\left/ {\vphantom {1 {\lambda_{1} }}}\right.\kern-0pt} \!\lower0.7ex\hbox{${\lambda_{1} }$}}} \right)}}{\Gamma }\left( {{\raise0.7ex\hbox{$2$} \!\mathord{\left/ {\vphantom {2 {\lambda_{1} }}}\right.\kern-0pt} \!\lower0.7ex\hbox{${\lambda_{1} }$}}} \right)}}} \right)^{{\lambda_{1} }} } \right)}}{{{\Gamma }\left( {{\raise0.7ex\hbox{$1$} \!\mathord{\left/ {\vphantom {1 {\lambda_{1} }}}\right.\kern-0pt} \!\lower0.7ex\hbox{${\lambda_{1} }$}}} \right)}} \\ & P_{2HEPD} = P\left( {t < t_{0} } \right) = \frac{{\gamma \left( {{\raise0.7ex\hbox{$1$} \!\mathord{\left/ {\vphantom {1 {\lambda_{1} }}}\right.\kern-0pt} \!\lower0.7ex\hbox{${\lambda_{1} }$}},\left( {\frac{{h_{1}^{{\lambda_{1} }} {\Gamma }\left( {{\raise0.7ex\hbox{$2$} \!\mathord{\left/ {\vphantom {2 {\lambda_{1} }}}\right.\kern-0pt} \!\lower0.7ex\hbox{${\lambda_{1} }$}}} \right)^{{\lambda_{1} }} }}{{\left( {{\Gamma }\left( {{\raise0.7ex\hbox{$1$} \!\mathord{\left/ {\vphantom {1 {\lambda_{1} }}}\right.\kern-0pt} \!\lower0.7ex\hbox{${\lambda_{1} }$}}} \right)} \right)^{{\lambda_{1} }} }}} \right)\left( {\frac{{\frac{{\lambda_{0}^{{{\raise0.7ex\hbox{$1$} \!\mathord{\left/ {\vphantom {1 {\lambda_{0} }}}\right.\kern-0pt} \!\lower0.7ex\hbox{${\lambda_{0} }$}}}} }}{{{\Gamma }\left( {{\raise0.7ex\hbox{$1$} \!\mathord{\left/ {\vphantom {1 {\lambda_{0} }}}\right.\kern-0pt} \!\lower0.7ex\hbox{${\lambda_{0} }$}}} \right)}}{\Gamma }\left( {{\raise0.7ex\hbox{$2$} \!\mathord{\left/ {\vphantom {2 {\lambda_{0} }}}\right.\kern-0pt} \!\lower0.7ex\hbox{${\lambda_{0} }$}}} \right)}}{{\frac{{\lambda_{1}^{{{\raise0.7ex\hbox{$1$} \!\mathord{\left/ {\vphantom {1 {\lambda_{1} }}}\right.\kern-0pt} \!\lower0.7ex\hbox{${\lambda_{1} }$}}}} }}{{{\Gamma }\left( {{\raise0.7ex\hbox{$1$} \!\mathord{\left/ {\vphantom {1 {\lambda_{1} }}}\right.\kern-0pt} \!\lower0.7ex\hbox{${\lambda_{1} }$}}} \right)}}{\Gamma }\left( {{\raise0.7ex\hbox{$2$} \!\mathord{\left/ {\vphantom {2 {\lambda_{1} }}}\right.\kern-0pt} \!\lower0.7ex\hbox{${\lambda_{1} }$}}} \right)}}} \right)^{{\lambda_{1} }} } \right)}}{{{\Gamma }\left( {{\raise0.7ex\hbox{$1$} \!\mathord{\left/ {\vphantom {1 {\lambda_{1} }}}\right.\kern-0pt} \!\lower0.7ex\hbox{${\lambda_{1} }$}}} \right)}} \\ \end{aligned}$$

Now the probability that ongoing operation is reported as IC when in fact it is moved to the alteration in shape parameter as follows

41 $$P_{in}^{2} = P(LCL \le D_{1} \le UCL|P_{2HEPD} )$$

42 $$P_{in}^{2} = \sum\nolimits_{{d_{1} = LCL + 1}}^{UCL} {\left( {\begin{array}{*{20}c} n \\ {d_{1} } \\ \end{array} } \right)\left( {P_{2HEPD} { }} \right)^{{d_{1} }} \left( {1 - P_{2HEPD} { }} \right)^{{n - d_{1} }} }$$

The ARL for shifted process due to its shape parameter denoted by ${ARL}_{2HEPD}$ is given as follows

43 $$ARL_{2HEPD} = \frac{1}{{1 - P_{in}^{2} }}$$

Now we utilize the accompanying calculation to register the Table of ARLs for offered CC using HEPD when its shape parameter is shifted.

1. Predefine the value of ARL and shape parameter designated by ${r}_{0} and{\lambda }_{0}$ respectively $.$
2. Determined the values of ${h}_{1},{k}_{1}$ according to the predetermined sample size $n$, for which ${ARL}_{0HEPD}$ in Eq. (33) is approach to ${r}_{0}.$
3. Use the values of ${h}_{1},{k}_{1}nd n$ attain in step 2 to determine the value of ${ARL}_{2HEPD}$ using Eq. (43) for assorted values of $w$.

#### Evaluation of proposed CC when both scale and shape parameters are shifted

In this section, we consider the scenario where both parameters of HEPD are shifted due to some extraneous variation. We suppose that the scale parameter is shifted as ${\alpha }_{1}=q{\alpha }_{0}$ and the shape parameter is shifted as ${\lambda }_{1}=w{\lambda }_{0}.$ The probability of an item failing before the predetermined period ${t}_{0}$ designated by ${P}_{3HEPD}$, can now be calculated as:

Rewrite Eq. (25)

$$P\left( {t < t_{0} } \right) = \frac{{\gamma \left( {{\raise0.7ex\hbox{$1$} \!\mathord{\left/ {\vphantom {1 {\lambda }}}\right.\kern-0pt} \!\lower0.7ex\hbox{${\lambda }$}},\left( {\frac{{h_{1}^{\lambda } {\Gamma }\left( {{\raise0.7ex\hbox{$2$} \!\mathord{\left/ {\vphantom {2 \lambda }}\right.\kern-0pt} \!\lower0.7ex\hbox{$\lambda $}}} \right)^{{\uplambda }} }}{{\left( {{\Gamma }\left( {{\raise0.7ex\hbox{$1$} \!\mathord{\left/ {\vphantom {1 \lambda }}\right.\kern-0pt} \!\lower0.7ex\hbox{$\lambda $}}} \right)} \right)^{{\uplambda }} }}} \right)\left( {\frac{{\mu_{0} }}{\mu }} \right)^{\lambda } } \right)}}{{{\Gamma }\left( {{\raise0.7ex\hbox{$1$} \!\mathord{\left/ {\vphantom {1 \lambda }}\right.\kern-0pt} \!\lower0.7ex\hbox{$\lambda $}}} \right)}}$$

As both parameters are shifted as ${\alpha }_{1}=q{\alpha }_{0} and {\lambda }_{1}=w{\lambda }_{0}$, so its mean level is also changed as ${\mu =\mu }_{1}$ where

44 $$\begin{aligned} & \mu_{1} = \frac{{\alpha_{1} \lambda_{1}^{{{\raise0.7ex\hbox{$1$} \!\mathord{\left/ {\vphantom {1 {\lambda_{1} }}}\right.\kern-0pt} \!\lower0.7ex\hbox{${\lambda_{1} }$}}}} }}{{{\Gamma }\left( {{\raise0.7ex\hbox{$1$} \!\mathord{\left/ {\vphantom {1 {\lambda_{1} }}}\right.\kern-0pt} \!\lower0.7ex\hbox{${\lambda_{1} }$}}} \right)}}{\Gamma }\left( {{\raise0.7ex\hbox{$2$} \!\mathord{\left/ {\vphantom {2 {\lambda_{1} }}}\right.\kern-0pt} \!\lower0.7ex\hbox{${\lambda_{1} }$}}} \right) \\ & P\left( {t < t_{0} } \right) = \frac{{\gamma \left( {{\raise0.7ex\hbox{$1$} \!\mathord{\left/ {\vphantom {1 {\lambda_{1} }}}\right.\kern-0pt} \!\lower0.7ex\hbox{${\lambda_{1} }$}},\left( {\frac{{h_{1}^{{\lambda_{1} }} {\Gamma }\left( {{\raise0.7ex\hbox{$2$} \!\mathord{\left/ {\vphantom {2 {\lambda_{1} }}}\right.\kern-0pt} \!\lower0.7ex\hbox{${\lambda_{1} }$}}} \right)^{{\lambda_{1} }} }}{{\left( {{\Gamma }\left( {{\raise0.7ex\hbox{$1$} \!\mathord{\left/ {\vphantom {1 {\lambda_{1} }}}\right.\kern-0pt} \!\lower0.7ex\hbox{${\lambda_{1} }$}}} \right)} \right)^{{\lambda_{1} }} }}} \right)\left( {\frac{{\mu_{0} }}{{\mu_{1} }}} \right)^{{\lambda_{1} }} } \right)}}{{{\Gamma }\left( {{\raise0.7ex\hbox{$1$} \!\mathord{\left/ {\vphantom {1 {\lambda_{1} }}}\right.\kern-0pt} \!\lower0.7ex\hbox{${\lambda_{1} }$}}} \right)}} \\ \end{aligned}$$

Substituting the values $$\mu_{1} , \alpha_{1} = q\alpha_{0} \,{\text{and}}\, \mu_{0} = \frac{{\alpha_{0} \lambda_{0}^{{1/\lambda_{0} }} }}{{\Gamma \left( {{\raise0.7ex\hbox{$1$} \!\mathord{\left/ {\vphantom {1 {\lambda_{0} }}}\right.\kern-0pt} \!\lower0.7ex\hbox{${\lambda_{0} }$}}} \right)}}\Gamma \left( {{\raise0.7ex\hbox{$2$} \!\mathord{\left/ {\vphantom {2 {\lambda_{0} }}}\right.\kern-0pt} \!\lower0.7ex\hbox{${\lambda_{0} }$}}} \right)$$ in the above equation

45 $$\begin{aligned} & P\left( {t < t_{0} } \right) = \frac{{\gamma \left( {{\raise0.7ex\hbox{$1$} \!\mathord{\left/ {\vphantom {1 {\lambda_{1} }}}\right.\kern-0pt} \!\lower0.7ex\hbox{${\lambda_{1} }$}},\left( {\frac{{h_{1}^{{\lambda_{1} }} {\Gamma }\left( {{\raise0.7ex\hbox{$2$} \!\mathord{\left/ {\vphantom {2 {\lambda_{1} }}}\right.\kern-0pt} \!\lower0.7ex\hbox{${\lambda_{1} }$}}} \right)^{{\lambda_{1} }} }}{{\left( {{\Gamma }\left( {{\raise0.7ex\hbox{$1$} \!\mathord{\left/ {\vphantom {1 {\lambda_{1} }}}\right.\kern-0pt} \!\lower0.7ex\hbox{${\lambda_{1} }$}}} \right)} \right)^{{\lambda_{1} }} }}} \right)\left( {\frac{{\frac{{\alpha_{0} \lambda_{0}^{{1/\lambda_{0} }} }}{{{\Gamma }\left( {{\raise0.7ex\hbox{$1$} \!\mathord{\left/ {\vphantom {1 {\lambda_{0} }}}\right.\kern-0pt} \!\lower0.7ex\hbox{${\lambda_{0} }$}}} \right)}}{\Gamma }\left( {{\raise0.7ex\hbox{$2$} \!\mathord{\left/ {\vphantom {2 {\lambda_{0} }}}\right.\kern-0pt} \!\lower0.7ex\hbox{${\lambda_{0} }$}}} \right)}}{{\frac{{q\alpha_{0} \lambda_{1}^{{1/\lambda_{1} }} }}{{{\Gamma }\left( {{\raise0.7ex\hbox{$1$} \!\mathord{\left/ {\vphantom {1 {\lambda_{1} }}}\right.\kern-0pt} \!\lower0.7ex\hbox{${\lambda_{1} }$}}} \right)}}{\Gamma }\left( {{\raise0.7ex\hbox{$2$} \!\mathord{\left/ {\vphantom {2 {\lambda_{1} }}}\right.\kern-0pt} \!\lower0.7ex\hbox{${\lambda_{1} }$}}} \right)}}} \right)^{{\lambda_{1} }} } \right)}}{{{\Gamma }\left( {{\raise0.7ex\hbox{$1$} \!\mathord{\left/ {\vphantom {1 {\lambda_{1} }}}\right.\kern-0pt} \!\lower0.7ex\hbox{${\lambda_{1} }$}}} \right)}} \\ & P_{3HEPD} = P\left( {t < t_{0} } \right) = \frac{{\gamma \left( {{\raise0.7ex\hbox{$1$} \!\mathord{\left/ {\vphantom {1 {\lambda_{1} }}}\right.\kern-0pt} \!\lower0.7ex\hbox{${\lambda_{1} }$}},\left( {\frac{{h_{1}^{{\lambda_{1} }} {\Gamma }\left( {{\raise0.7ex\hbox{$2$} \!\mathord{\left/ {\vphantom {2 {\lambda_{1} }}}\right.\kern-0pt} \!\lower0.7ex\hbox{${\lambda_{1} }$}}} \right)^{{\lambda_{1} }} }}{{\left( {{\Gamma }\left( {{\raise0.7ex\hbox{$1$} \!\mathord{\left/ {\vphantom {1 {\lambda_{1} }}}\right.\kern-0pt} \!\lower0.7ex\hbox{${\lambda_{1} }$}}} \right)} \right)^{{\lambda_{1} }} }}} \right)\left( {\frac{{\frac{{\lambda_{0}^{{1/\lambda_{0} }} }}{{{\Gamma }\left( {{\raise0.7ex\hbox{$1$} \!\mathord{\left/ {\vphantom {1 {\lambda_{0} }}}\right.\kern-0pt} \!\lower0.7ex\hbox{${\lambda_{0} }$}}} \right)}}{\Gamma }\left( {{\raise0.7ex\hbox{$2$} \!\mathord{\left/ {\vphantom {2 {\lambda_{0} }}}\right.\kern-0pt} \!\lower0.7ex\hbox{${\lambda_{0} }$}}} \right)}}{{\frac{{q\lambda_{1}^{{1/\lambda_{1} }} }}{{{\Gamma }\left( {{\raise0.7ex\hbox{$1$} \!\mathord{\left/ {\vphantom {1 {\lambda_{1} }}}\right.\kern-0pt} \!\lower0.7ex\hbox{${\lambda_{1} }$}}} \right)}}{\Gamma }\left( {{\raise0.7ex\hbox{$2$} \!\mathord{\left/ {\vphantom {2 {\lambda_{1} }}}\right.\kern-0pt} \!\lower0.7ex\hbox{${\lambda_{1} }$}}} \right)}}} \right)^{{\lambda_{1} }} } \right)}}{{{\Gamma }\left( {{\raise0.7ex\hbox{$1$} \!\mathord{\left/ {\vphantom {1 {\lambda_{1} }}}\right.\kern-0pt} \!\lower0.7ex\hbox{${\lambda_{1} }$}}} \right)}} \\ \end{aligned}$$

Now the probability for IC is given by

46 $$P_{in}^{3} = P(LCL \le D_{1} \le UCL|P_{3HEPD} )$$

47 $$P_{in}^{3} = \mathop \sum \limits_{{d_{1} = LCL + 1}}^{UCL} \left( {\begin{array}{*{20}c} n \\ {d_{1} } \\ \end{array} } \right)\left( {P_{3HEPD} { }} \right)^{{d_{1} }} \left( {1 - P_{3HEPD} { }} \right)^{{n - d_{1} }}$$

The ARL for shifted process due to change in both parameters denoted by ${ARL}_{3HEPD}$ is given as follows

48 $$ARL_{3HEPD} = \frac{1}{{1 - P_{in}^{3} }}$$

Now, we use the subsequent calculation to register the Table of ARLs for suggested CC when both parameters of HEPD are shifted.

1. Fix the values of ARL and shape parameter designated by ${r}_{0} and{\lambda }_{0}$ respectively $.$
2. Finding the values of ${h}_{1},{k}_{1}$ according to the predetermined sample size $n$, for which ${ARL}_{0HEPD}$ in Eq. (33) is approach to ${r}_{0}.$
3. Use the values of ${h}_{1},{k}_{1}nd n$ attain in step 2 to determine the value of ${ARL}_{3HEPD}$ using Eq. (48) for different combinations of $q and w$.

## Results discussion

In this section, we discuss the results obtained from two life data distributions. The values of ${ARL}_{1HND},$ ${ARL}_{1HEPD}, {ARL}_{2HEPD}$ and ${ARL}_{3HEPD}$ for various shifts in scale and shape parameters are given in Tables 1, 2, 3, 4, 5, 6 and 7.

**Table 1:** The ARL values of proposed CC using HND.

	$n=15$	$n=25$	$n=15$	$n=25$
Control coefficient $\to$	$k=2.837$	$k=2.866$	$k=3.085$	$k=3.103$
Truncated constant $\to$	$h=0.3058$	$h=0.6284$	$h=0.2961$	$h=0.5565$
$c$	${ARL}_{1HND}$	${ARL}_{1HND}$	${ARL}_{1HND}$	${ARL}_{1HND}$
1	300.26	300.12	370.03	370.10
0.95	216.20	198.25	265.61	234.66
0.93	188.95	161.79	231.81	190.90
0.90	153.80	116.63	188.29	138.00
0.85	108.03	65.48	131.73	78.54
0.80	74.89	36.24	90.90	44.12
0.75	51.21	20.06	61.83	24.72
0.70	34.54	11.23	41.45	13.92
0.60	15.11	3.81	17.84	4.67
0.50	6.35	1.64	7.33	1.89
0.30	1.34	1.00	1.41	1.00
0.10	1.00	1.00	1.00	1.00

**Table 2:** The ARL values of Proposed CC using HEPD when its scale parameter is shifted using ${r}_{o}=300 \mathrm{and} n=15$.

	$\lambda =1$	$\lambda =2$	$\lambda =3$	$\lambda =4$
Control coefficient $\to$	${k}_{1}=2.775$	${k}_{1}=2.837$	${k}_{1}=2.884$	${k}_{1}=2.923$
Truncated constant $\to$	${h}_{1}=0.2141$	${h}_{1}=0.3058$	${h}_{1}=0.429$	${h}_{1}=0.4493$
$q$	${ARL}_{1HEPD}$	${ARL}_{1HEPD}$	${ARL}_{1HEPD}$	${ARL}_{1HEPD}$
1	300.58	300.26	300.69	300.67
0.90	163.25	153.80	141.08	140.37
0.80	84.92	74.89	62.64	61.89
0.70	42.16	34.54	26.29	25.73
0.60	19.95	15.11	10.52	10.16
0.50	9.05	6.35	4.15	3.94
0.40	4.02	2.70	1.79	1.68
0.30	1.88	1.34	1.07	1.04
0.20	1.11	1.01	1.00	1.00
0.10	1.00	1.00	1.00	1.00

**Table 3:** The ARL values of proposed CC using HEPD when its scale parameter is shifted using ${r}_{o}=370 \mathrm{and} n=15$.

	$\lambda =1$	$\lambda =2$	$\lambda =3$	$\lambda =4$
Control Coefficient $\to$	${k}_{1}=3.081$	${k}_{1}=3.085$	${k}_{1}=3.091$	${k}_{1}=3.116$
Truncated Constant $\to$	${h}_{1}=0.2067$	${h}_{1}=0.2961$	${h}_{1}=0.417$	${h}_{1}=0.4368$
$q$	${ARL}_{1HEPD}$	${ARL}_{1HEPD}$	${ARL}_{1HEPD}$	${ARL}_{1HEPD}$
1	370.40	370.03	370.24	370.23
0.90	199.63	188.29	172.51	171.71
0.80	102.87	90.90	75.89	75.05
0.70	50.48	41.45	31.45	30.82
0.60	23.53	17.84	12.35	11.95
0.50	10.45	7.33	4.74	4.51
0.40	4.51	3.01	1.95	1.83
0.30	2.03	1.41	1.10	1.06
0.20	1.14	1.01	1.00	1.00
0.10	1.00	1.00	1.00	1.00

**Table 4:** The ARL values of proposed CC using HEPD when its shape parameter is shifted using ${r}_{o}=300 ,370 and n=15$.

	$\lambda =2$	$\lambda =4$	$\lambda =2$	$\lambda =4$
Control Coefficient $\to$	${k}_{1}=2.837$	${k}_{1}=2.923$	${k}_{1}=3.085$	${k}_{1}=3.116$
Truncated Constant $\to$	${h}_{1}=0.3058$	${h}_{1}=0.4493$	${h}_{1}=0.2961$	${h}_{1}=0.4368$
$w$	${ARL}_{2HEPD}$	${ARL}_{2HEPD}$	${ARL}_{2HEPD}$	${ARL}_{2HEPD}$
1	300.26	300.674	370.03	370.23
0.99	242.76	150.281	298.53	183.94
0.98	197.05	77.037	241.79	93.64
0.95	108.07	12.547	131.67	14.82
0.90	43.41	1.606	52.17	1.74
0.85	19.66	1.003	23.26	1.01
0.80	10.10	1.00	11.74	1.00
0.75	5.90	1.00	6.74	1.00
0.70	3.90	1.00	4.37	1.00
0.65	2.88	1.00	3.18	1.00
0.60	2.35	1.00	2.56	1.00
0.50	2.02	1.00	2.17	1.00

**Table 5:** The ARL values of proposed CC using HEPD when both shape and scale parameters are shifted using ${r}_{o}=300, 370 \mathrm{and} n=15$.

	$\lambda =2$	$\lambda =4$	$\lambda =2$	$\lambda =4$
Control Coefficient $\to$	${k}_{1}=2.837$	${k}_{1}=2.923$	${k}_{1}=3.085$	${k}_{1}=3.116$
Truncated Constant $\to$	${h}_{1}=0.3058$	${h}_{1}=0.4493$	${h}_{1}=0.2961$	${h}_{1}=0.4368$
$(w,q)$	${ARL}_{3HEPD}$	${ARL}_{3HEPD}$	${ARL}_{3HEPD}$	${ARL}_{3HEPD}$
(1, 1)	300.26	300.674	370.03	370.23
(0.99, 0.99)	227.69	139.907	279.83	171.13
(0.98, 0.98)	173.52	67.082	212.64	81.41
(0.95, 0.95)	79.33	9.356	96.28	10.98
(0.90, 0.90)	24.32	1.267	28.93	1.34
(0.85, 0.85)	8.91	1.00	10.34	1.00
(0.80, 0.80)	4.00	1.00	4.51	1.00
(0.75, 0.75)	2.22	1.00	2.43	1.00
(0.70, 0.70)	1.51	1.00	1.60	1.00
(0.65, 0.65)	1.21	1.00	1.26	1.00
(0.60, 0.60)	1.09	1.00	1.11	1.00
(0.50, 0.50)	1.02	1.00	1.02	1.00

**Table 6:** Comparison of ARLs when ${r}_{o}=370 \mathrm{and} n=15$.

	Proposed HEPD based chart when both parameters are shifted using $\lambda =$ 4		Proposed HEPD when shape parameter is shifted using $\lambda =$ 4	Proposed HEPD when scale parameter is shifted using $\lambda =$ 4	Proposed chart based on HND	Existing chart proposed by Ref. ^8^
Control Coefficient $\to$	${k}_{1}=3.116$		${k}_{1}=3.116$	${k}_{1}=3.116$	${k}_{1}=3.085$	${k}_{1}=3.081$
Truncated constant $\to$	${h}_{1}=$ 0.4368		${h}_{1}=0.4368$	${h}_{1}=0.4368$	${h}_{1}=$ 0.2961	${h}_{1}=0.2067$
Shift $(w,q)$	${ARL}_{3HEPD}$	Shift	${ARL}_{2HEPD}$	${ARL}_{1HEPD}$	${ARL}_{1HND}$	${ARL}_{1}$
(1, 1)	370.23	1	370.23	370.23	370.03	370.4
(0.99, 0.99)	171.13	0.99	183.90	343.70	346.60	348.90
(0.98, 0.98)	81.41	0.98	93.64	318.90	324.50	328.40
(0.95, 0.95)	10.98	0.95	14.82	253.90	265.60	273.40
(0.90, 0.90)	1.34	0.90	1.74	171.70	188.29	199.60
(0.85, 0.85)	1.00	0.85	1.01	114.40	131.70	144.20
(0.80, 0.80)	1.00	0.80	1.00	75.04	90.90	102.09
(0.75, 0.75)	1.00	0.75	1.00	48.46	61.83	72.52
(0.70, 0.70)	1.00	0.70	1.00	30.82	41.45	50.47
(0.65, 0.65)	1.00	0.65	1.00	19.31	27.39	34.68
(0.60, 0.60)	1.00	0.60	1.00	11.95	17.84	23.52
(0.50, 0.50)	1.00	0.50	1.00	4.51	7.33	10.45
(0.30, 0.30)	1.00	0.30	1.00	1.06	1.41	2.02
(0.10, 0.10)	1.00	0.10	1.00	1.00	1.0	1.0

**Table 7:** Simulated data.

${D}_{1}$	${D}_{1}$	${D}_{1}$	${D}_{1}$
3	4	3	5
1	1	6	5
2	5	6	2
2	4	3	7
3	4	7	10
6	4	8	4
7	2	3	5
1	5	5	4
4	8	3	4
4	1	3	6

**Figure 1:**
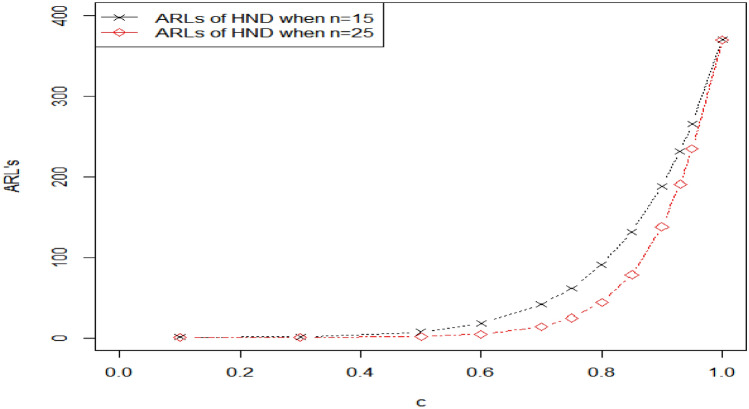
Graph of ARLs for HND using ${r}_{o}=370 \mathrm{and} n=15 \mathrm{and} 25$.

Table 1 shows the values of ARLs for HND for various scale parameter shifts and sample sizes when the ARLs are 300,370. The shifted constant has a range of 1 to 0.1. when the value of shifted constant $c=1,$ the corresponding value of ARL is closed to predefine ${r }_{0}$. Table 1 displays decreasing behavior in ARLs as the shifted constant $c$ increases. We also observe that the performance of the given charting structure is improved for a larger sample size. For example, when ${r}_{0}=370, k=3.085, h=0.2961, q=0.93$ and $n=15$ the calculated value of out-of-control (OOC) ${ARL}_{1HND}$ is 231.81, indicating that on average, it would take 231.81 subgroups to be sampled before the CC detects an OOC condition and for larger sample size say $n=25,$ ${r}_{0}=370, k=3.013, , h=0.5565, q=0.93$ the OOC ${ARL}_{1HND}$ is reduced to 190.90, implying that on average, only 190.90 subgroups would be sampled before the CC signals an OOC condition. The values of ${ARL}_{1HND}$ using sample sizes 15 and 25 are also plotted in Fig. 1, which shows that a larger sample size leads to smaller ARLs, thus demonstrating an improvement.

**Figure 2:**
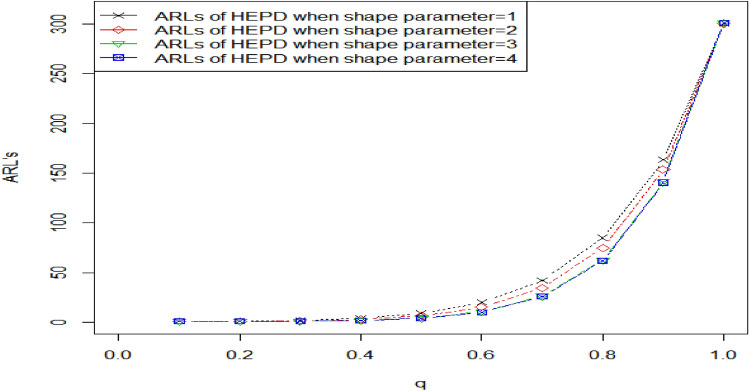
Graph of ARLs for HEPD when scale parameter is shifted using ${r}_{o}=300 \mathrm{and} n=$ 15.

**Figure 3:**
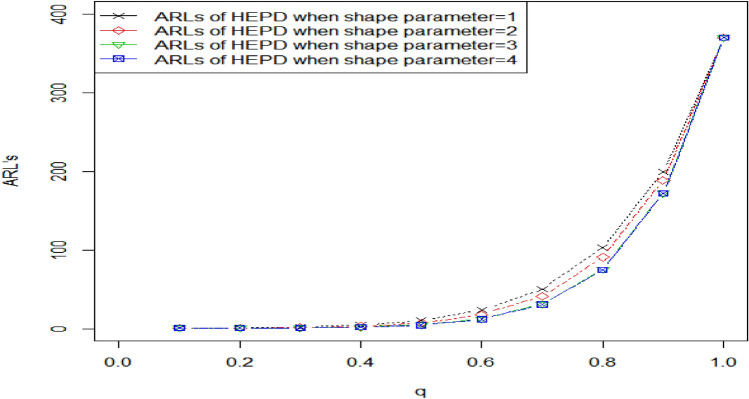
Graph of ARLs for HEPD when scale parameter is shifted using ${r}_{o}=370 \mathrm{and} n=$ 15.

Tables 2, 3 show the OOC ${ARL}_{1HEPD}$ values of HEPD when its scale parameter is shifted. As we know that the range of shape parameter $\lambda >0,$ we considered different values of $\lambda$ (namely, $\lambda$ = 1, 2, 3, 4) for the construction of Tables 2, 3. Such parametric values have been used by several researchers like in Refs. ^8^, ^23,24^. The values of OOC ${ARL}_{1HEPD}$ are calculated using various shifts $\left(q\right)$ in scale parameter with ${r}_{o}=300 \mathrm{and} 370 \mathrm{and} n=15$, these values are also plotted in Figs. 2, 3. The in-control ARLs are presented for the shifted values $\left(q=1\right)$.We see the decline inclination in ARLs by changing the value of shifted constant $\left(q\right)$ in these Tables from 1 to 0.1. Upon examining Tables 2, 3, it becomes apparent that as the value of shape parameter $\lambda$ increases, the effectiveness of the proposed chart improves even with the same amount of shift in the scale parameter. We also see that larger diversions in parameters have been detected more quickly. Diversion refers to any significant deviation or change in the process from its expected behavior. The ARL values in CCs are used to measure the performance of a chart in detecting such deviations. The larger values of shifted constantly are more sensitive to detecting process deviations. The larger the value of the constant, the higher the probability of detecting a deviation in the process, which means that the ARL values are smaller. By detecting deviations more quickly, the control chart can help prevent the occurrence of defects or other undesirable outcomes. For example, In Table 3 when ${r}_{0}=370, n=15, \lambda =3 , {h}_{1}=0.417,{k}_{1}=3.091$ and the shifted amount $q=0.90$, the value of OOC $A{ARL}_{1HEPD}$ is 172.51. it means that on average, it takes 172.51 subgroups to be sampled before the CC signals an OOC condition, and for larger shifts say $q=0.80$, we notice the OOC ${ARL}_{1HEPD}$ is 75.89. it means that on average, it takes just 75.89 subgroups to be sampled before the CC signals an OOC condition. Similarly, for $q=0.70, ARL=31.45,$ for $q=0.30, ARL=1.10.$

**Figure 4:**
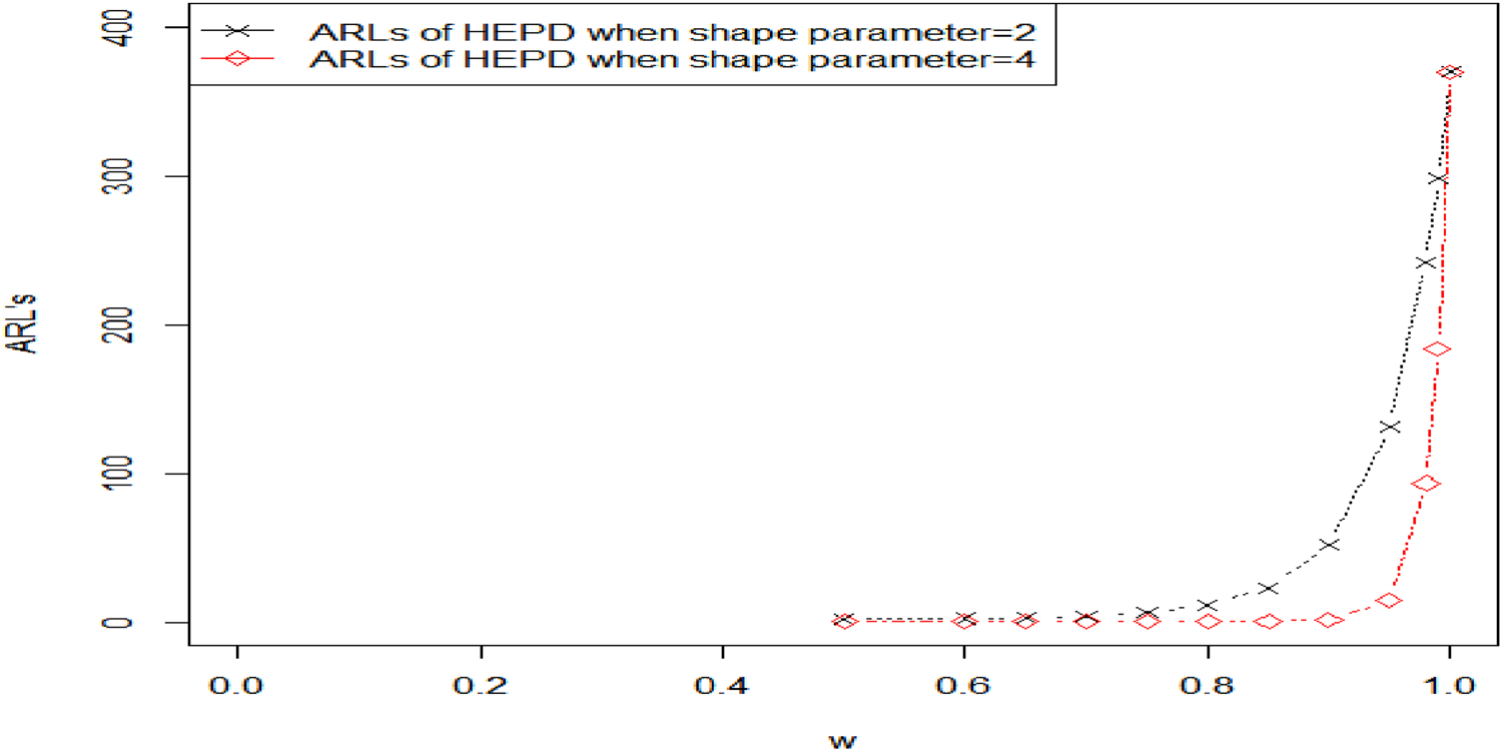
Graph of the ARLs for HEPD when shape parameter is shifted, using ${r}_{o}=370,n=15 \mathrm{and} \lambda =2 \mathrm{and} 4$.

**Figure 5:**
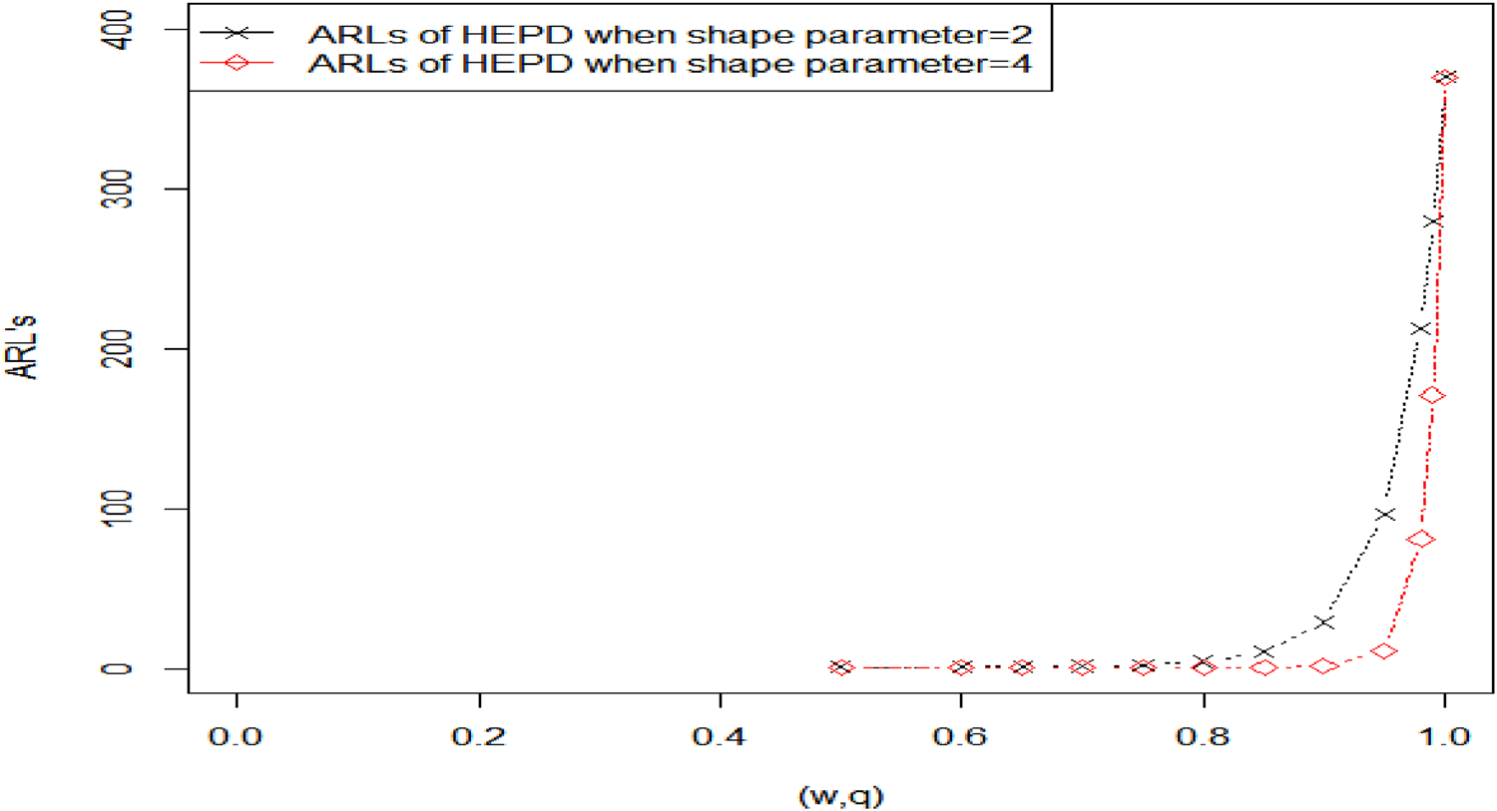
Graph of the ARLs for HEPD when both the parameters are shifted, using ${r}_{o}=370,n=15 \mathrm{and} \lambda =2 \mathrm{and} 4$.

Table 4 displays the OOC ${ARL}_{2HEPD}$ values of HEPD for different shifts in the shape parameter. It is notable that as the process shift becomes larger, the value of ${ARL}_{2HEPD}$ decreases sharply. Additionally, when λ has a larger value, the outcomes show significant improvement in terms of smaller ${ARL}_{2HEPD}$. These findings are visually represented in Fig. 4. Table 5 presents the OOC ${ARL}_{3HEPD}$ values of HEPD for different shifts in both the scale and shape parameters. These results are also plotted in Fig. 5. It can be observed that the suggested charting structure is greatly enhanced when both parameters are shifted simultaneously. ${r}_{0}=370, \lambda =2 , {k}_{1}=2.807, {h}_{1}=0.2961,\left(w,q\right)=\left(\mathrm{0.9,0.9}\right)$ and $n=15$ the value of ${ARL}_{3HEPD}=28.93.$ In contrast, when only the scale parameter is shifted (as shown in Table 3), ${ARL}_{1HEPD}$ is188.29 with $q=0.9, \lambda =2,$ ${h}_{1}=0.2961, {k}_{1}=3.085,{r}_{o}=370 \mathrm{and} n=15$. Similarly, when only the shape parameter is shifted (as shown in Table 4), the value of ${ARL}_{2HEPD}$ is 52.17 with $w = 0.90$ and the remaining values are kept the same.

## Advantages of proposed control charts

This section evaluates the benefits of the presented control charts using HND and HEPD compared to the existing CC recommended by Ref. ^8^. Furthermore, the superiority of ACC for HEPD is examined over the ACC for HND under TTLT. The ability of the suggested CC is assessed in terms of OOC ARL. As previously mentioned in the Results Discussion Section, the performance of the proposed chart improves with larger values of $\lambda$. Therefore, in the comparison section, we have set $\lambda =4$ for the proposed HEPD-based chart for the purpose of comparison. We have tabulated the OOC ARLs for various shifts in the scale parameter, shape parameter, and both parameters simultaneously for the proposed HEPD. We have also included the OOC ARLs for other proposed HND and OOC ARLs recommended by Ref. ^8^ in Table 6. A smaller value of OOC ARL indicates a better ability to detect the OOC condition.

### Comparison of the Attribute control chart for HEPD versus the attribute control chart for exponential distribution (ED) under TTLT

**Figure 6:**
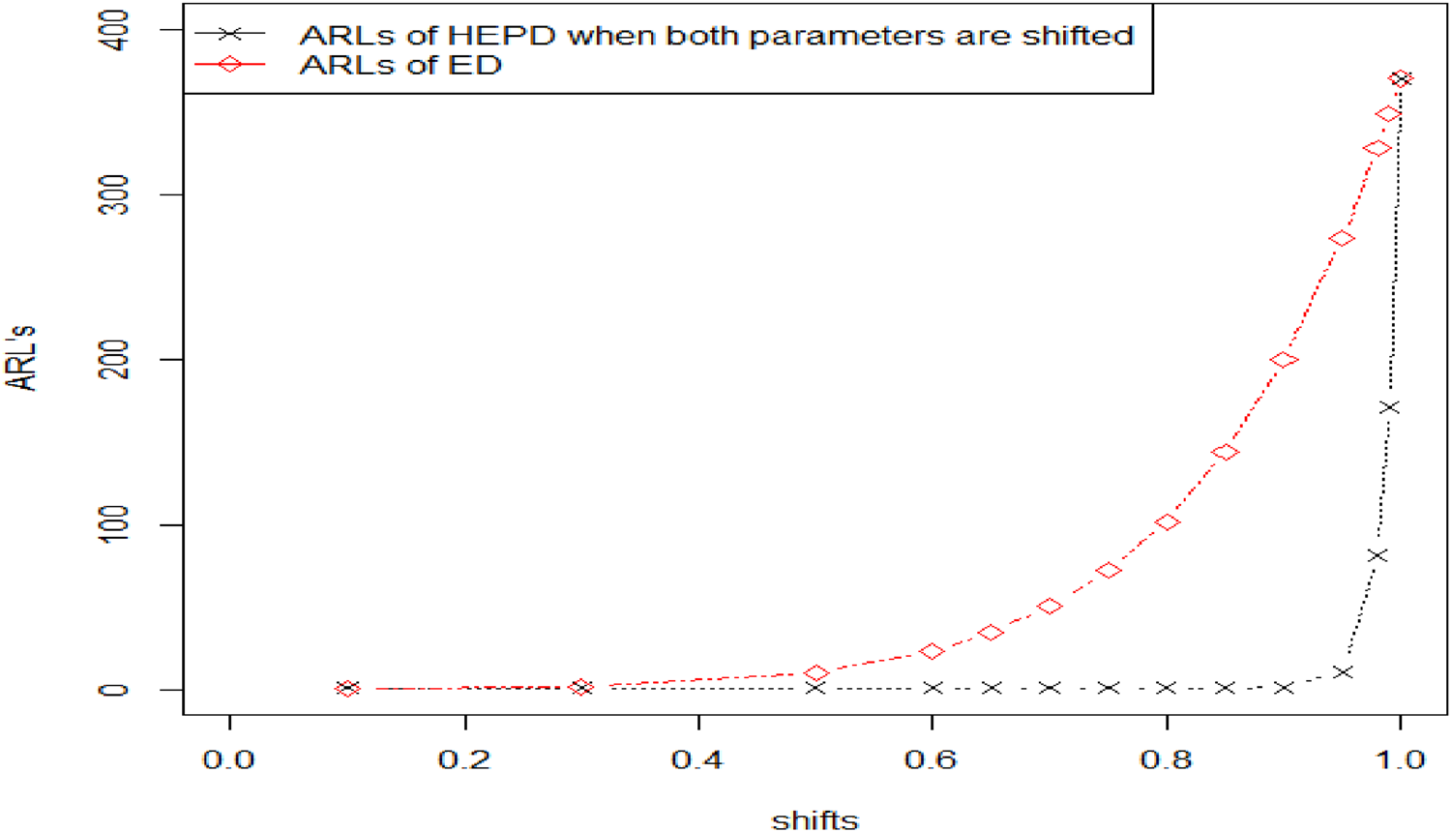
Graph of ARLs for HEPD verses ED when ${r}_{0}=370,n=15$.

Here, we discuss the dominance of the planned chart by comparing it with the chart suggested by Ref. ^8^. The suggested chart is the generalization of ACC for ED under TTLT. The suggested control chart is converted to Ref. ^8^ when its shape parameter $\lambda =1.$ The values of OOC ARLs using ACC for ED and the proposed chart using HEPD for the case scale parameter, shape parameter and both the parameters are shifted under TTLT for sample size 15 and ${r}_{0}=370$ are presented in Table 6. We perceive the smaller OOC ARLs of the suggested CC for sundry shifts in all cases of shifted parameters. For example, when ${r}_{0}=370, n=15, {h}_{1}=0.4368, \lambda =4 ,{k}_{1}=3.116$ and $q=0.98$ the value of OOC ${ARL}_{1HEPD}$ is 343.70, ${ARL}_{2HEPD}$ is 183.90 and ${ARL}_{3HEPD}$ is 171.13, and for ^8^ it was 348.90 when ${r}_{0}=370, n=15,{h}_{1}=0.2067, {k}_{1}=3.081and q=0.98.$ The above result indicates that recommended CC is more powerful in pointing out the smaller shifts in process parameters. Figure 6 also demonstrates the contrast between these two distributions.

### Comparison of the attribute control chart for HEPD verses attribute control chart for half normal distribution under TTLT

**Figure 7:**
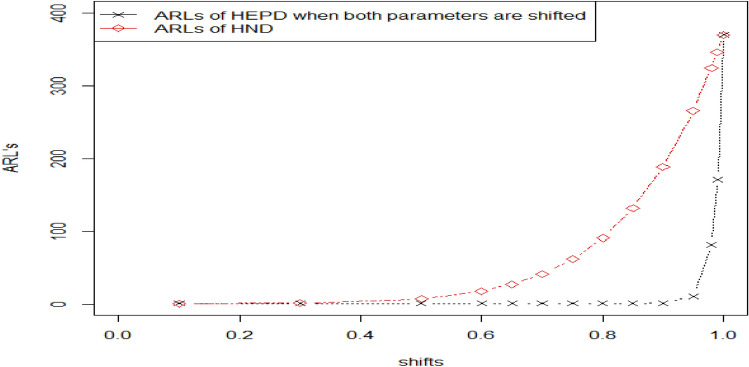
Graph of ARLs for HEPD verses HND when ${r}_{0}=370,n=15$.

Here we examine the benefits of recommended HEPD chart as compared to the other proposed ACC for HND under TTLT. The proposed HEPD chart is converted to the ACC for HND under TTLT when its shape parameter $\lambda =2$. So, we can utter that suggested chart is the extension of the HND under TTLT. The OOC ARL values of HND under TTLT are reported in Tables 1 and 6 for different levels of shift. We observe the smaller ARLs of the recommended HEPD chart for different shifts. For example,, when ${r}_{0}=370, \lambda =4,n=15,{h}_{1}=0.4368,{k}_{1}=3.116$ and $q=0.98,$ the OOC value for HEPD based chart are as follows: ${ARL}_{1HEPD}=318.90, {ARL}_{2HEPD}=93.64 and {ARL}_{3HEPD}=81.41$*,* whereas for HND, it is 324.50 when ${r}_{0}=370,n=15, {h}_{1}=0.2961,{k}_{1}=3.085 and q=0.98.$ Thus, the proposed charting scheme using HEPD is more effective in identifying minor shifts in process parameters compared to the ACC of HND under TTLT. The comparison of these two distributions can also be seen in Fig. 7.

### Comparison of the Attribute control chart for half normal distribution verses attribute control chart for exponential distribution under TTLT

**Figure 8:**
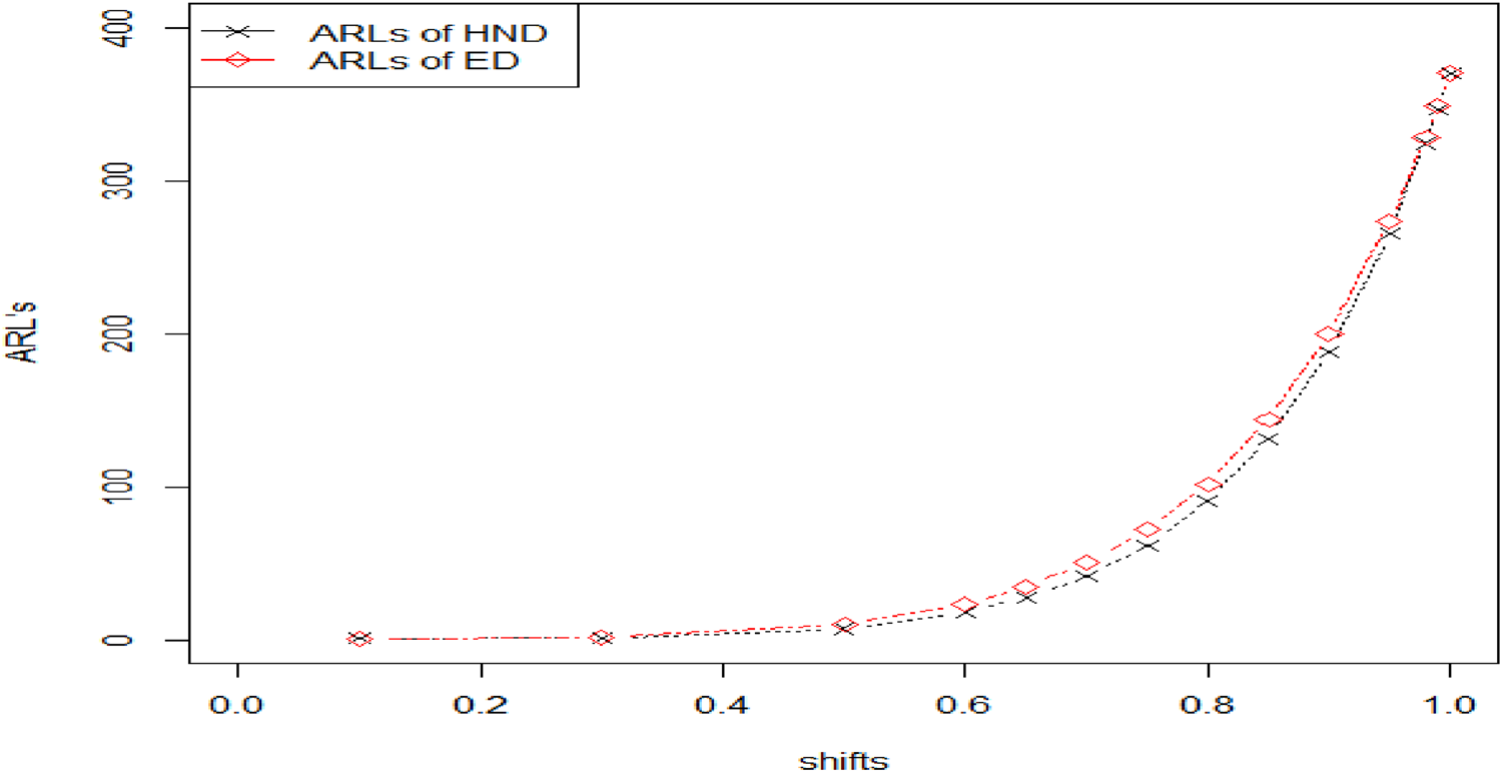
Graph of ARLs for HND verses ED when ${r}_{0}=370,n=15$.

Here, we confer the advantages of proposed ACC for HND by comparing it to the ACC for ED under TTLT proposed by Ref. ^8^. Both distributions are special cases of HEPD. If $\lambda =1,$ HEPD convert to ED and for $\lambda =2,$ HEPD reduce to HND. ARLs values of both distributions under TTLT are presented in Table 6 for different shifts. We notice that HND has smaller ARL values when compared to ED under TTLT for different shifts. For example when ${r}_{0}=370, n=15, {h}_{1}=0.2961,{k}_{1}=3.085$ and $q=0.80$ the value of OOC ARL for HND is 90.90 and for ED it was 102.87 when ${r}_{0}=370, n=15, {h}_{1}=0.2067 ,{k}_{1}=3.081 and q=0.80.$ Thus, HND has competency to search the minor shifts in parameter earlier than ED under TTLT. The contrast between these two distributions can also be observed in Fig. 8.

## Simulation study

**Figure 9:**
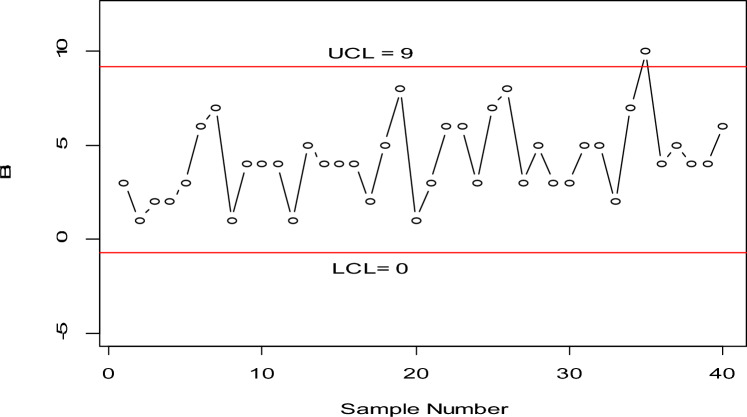
Graph of the proposed CC for simulated data.

This section presents a simulation study to verify the effectiveness of the proposed charting scheme. To conduct the study, we generated 20 observations of subgroup size 15 from HEPD with scale parameter $\alpha =1$ and shape parameter $\lambda =2$, assuming the process is in a normal state with ${r}_{0}=300$. We then introduced a shift in the manufacturing process and generated another 20 observations of subgroup size 15 from HEPD with the shifted scale parameter ${\alpha }_{1}=q{\alpha }_{0}$, $q=0.60$. The truncated time ${t}_{0}={(h}_{1}*\mu )$, calculated as using parameter $\alpha =1,\lambda =2$ and ${r}_{0}=300$, gives the value of ${h}_{1}$ from Table 2 as ${h}_{1}=0.3058$, and value of $\mu$ using Eq. (8) against parameter $\alpha =1,\lambda =2$ calculated as $\mu =0.7978$. Hence the value of truncated time ${t}_{0}={(h}_{1}*\mu )=0.3058*0.7978=0.244.$ The number of failure items that occurred before the truncated time ${t}_{0}$ in each subgroup is denoted as ${D}_{1}$ and recorded in Table 7. By using these values, we calculated the $LCL=0$ and $UCL=9$, using Eqs. (29) and (30) and with ${k}_{1}=2.837$ from Table 2 based on the parameter $\alpha =1,\lambda =2$ and ${r}_{0}=300$. These observations are also plotted in Fig. 9 which demonstrates the procedure is OOC at the 35th observation, which is the 15th observation after the shift. The same ARL value is reported in Table 2. Subsequently, it is evidently demonstrating that the proposed CC identifies the shifts proficiently.

## Real life examples

In this section, we will discuss three real data-based examples to illustrate the effectiveness of their proposed idea. The first two examples are based on HEPD, while the third example is based on HND. Our aim is to improve the generalizability of the results and provide a more comprehensive assessment of the evaluation of the proposed control charts.

### Application I

**Figure 10:**
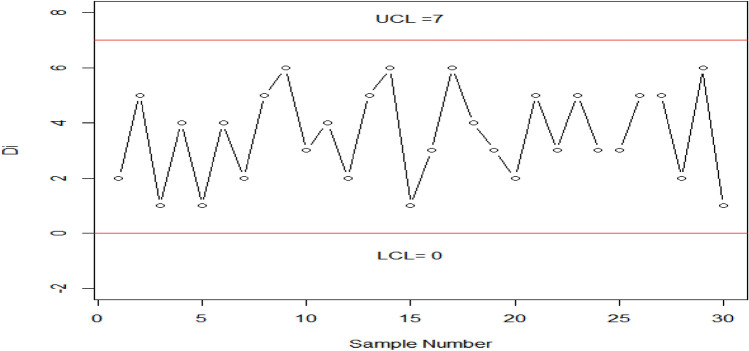
Graph of proposed CC using HEPD with real life data Application I.

The effectiveness of suggested CC is also judged with the assistance of real-life examples. The data on the 90% stress level of Kevlar 49/epoxy stands comprised of one hundred and one observations that were exposed to enduring pressure until their breakdown time. The same dataset has been discussed by numerous authors, like Refs.^25,26^. It is noted that the life time of one hundred and one observations pursues the HEPD with $\lambda =0.8815$ and $\alpha =0.9689$. The average life of the data is given as ${\mu }_{0}=1.025$ h ^20^. It is assuming that ${r}_{0}=370,n=15$ and ${h}_{1}=0.1877$. Now, by utilizing Eq. (26), the value of ${P}_{0}$ using the values $\lambda =0.8815, {h}_{1}=0.1877$ we have ${P}_{0}=0.1867.$ The LCL and UCL using Eqs. (27, 28) are 0 and 7, respectively when $n=15,{P}_{0}=0.1867 \mathrm{and} {k}_{1}=3.02$.The truncated time ${t}_{0}={h}_{1}*{\mu }_{0}=0.1877*1.025=0.1924 \mathrm{h}$. The working procedure of the intended chart is as follows:

- *Stage 1* we draw an arbitrary sample of size fifteen at each subgroup from HEPD using scale parameter $\alpha =0.9689$ and shape parameter $\lambda =0.8815$, and put them on truncated time ${t}_{0}=0.19244$ hours. Count the number of failure items ($D$) during the test which is also plotted in Fig. 10.
- *Stage 2* we determine the working procedure as IC if the number of failure items is between $0 and 7 (0\le D\le 7)$. If the number falls outside this range, the process is deemed OOC.

### Application II

**Figure 11:**
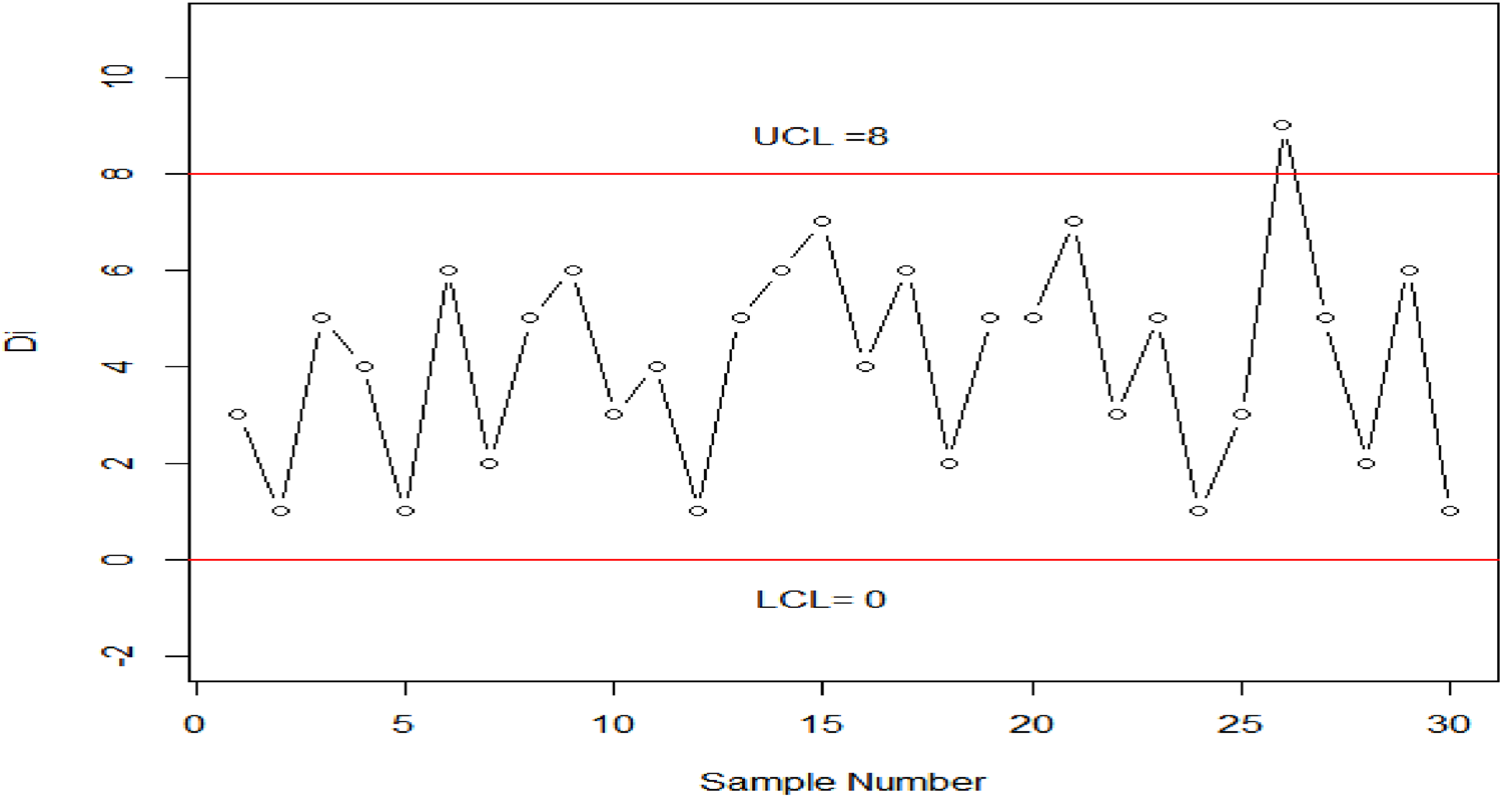
Graph of proposed CC using HEPD with real life data Application II.

The effectiveness of the proposed CC is also evaluated using real-life data obtained from Gui (2013b), which consists of plasma ferritin cluster measurements from 202 athletes gathered at the Australian Institute of Sport. This dataset has been studied by several authors, including Refs. ^27–29^. The data set follows the HEPD with a mean of 76.88 plasma ferritin and a standard deviation of 47.50 plasma ferritin. The scale parameter $\alpha$ is known to be $97.1311$ and the shape parameter $\lambda$ is 2.5109. Assuming ${r}_{0}=300,n=15$ and ${h}_{1}=0.3274$, we can obtain the value of ${P}_{0}$ using Eq. (26) when $\lambda =2.5109\mathrm{ and }{h}_{1}=0.3274$, as ${P}_{0}=0.193.$ The LCL and UCL using Eqs. (27, 28) are 0 and 8 respectively, when $n=15,{P}_{0}=0.193 \mathrm{and} {k}_{1}=3.2$. The truncated time ${t}_{0}={h}_{1}*{\mu }_{0}=0.3274*76.88=25.17 \mathrm{plasma ferritin}$. The following steps describe how the chart works:

- *Stage 1* A sample of 15 items is randomly selected from the HEPD distribution with a scale parameter $\alpha =97.1311$ and a shape parameter $\lambda =2.5109$. These items are then placed on a truncated time ${t}_{0}=25.17 \mathrm{plasma ferritin}.$ The number of failed items (D) is counted during the test, and the results are plotted in Fig. 11.
- *Stage 2* The process is declared as IC if $0\le D\le 8$; otherwise, it is considered as OOC.

### Application III

**Figure 12:**
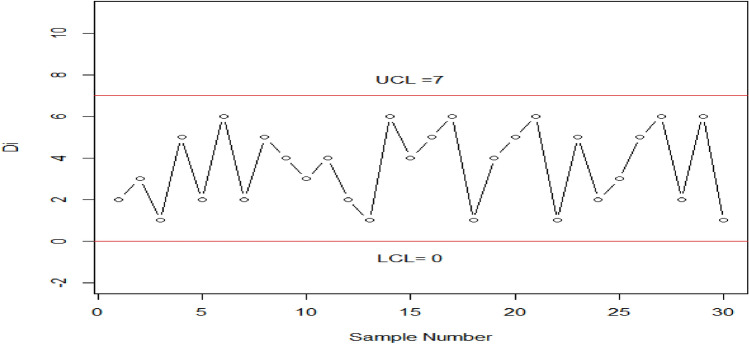
Graph of proposed CC using HND with real life data Application II.

In this section, we assess the efficacy of the suggested CC using an 86-sample dataset acquired from the mining department. The data measures the concentration of zinc (Zn) in soil samples and was previously used in a study conducted by Ref.^26^. The soil data is known to follow the HND with a mean of 96.72 Zn, standard deviation of 148.4 Zn and scale parameter α = 176.44 Zn. we suppose that ${r}_{0}=300, n=15,$ and $h=0.3058$. Now, by utilizing Eq. (11), the value of ${P}_{0}$ when $h=0.3058$ is ${P}_{0}=0.1928.$ The LCL and UCL using Eqs. (12, 13) are 0 and 7 respectively when $n=15,{P}_{0}=0.1928 \mathrm{and} k=2.837$.The truncated time ${t}_{0}=h*{\mu }_{0}=0.3058*96.72=29.58 \mathrm{Zn}$. The working procedure of the intended chart is as follows; firstly, we randomly select a subgroup of size fifteen from HND using scale parameter $\alpha =176.44$ and place them on the truncated time, ${t}_{0}=29.58$. We then count the number of failure items (D) during the test and plot the results in Fig. 12. If the plotted values fall within the range of $0 to 7 (0\le D\le 7)$ we declare the working procedure as IC. Otherwise, the process is recognized as OOC.

## Concluding remarks

In this paper, we recommend two types of ACC based on HND and HEPD, utilizing TTLT. ARL Tables have been constructed by using HND with different levels of shift in scale parameter, and for HEPD, we have constructed tables of ARL using shifts in three cases: firstly, we consider shifts in scale parameter only, after that, we introduce shifts in shape parameter; and lastly, we have constructed tables when both parameters are shifted simultaneously under TTLT. The results of ARLs based on HEPD have shown smaller ARLs when we compare them with ED and HND based ACC under TTLT. Additionally, we have made a comparison of the proposed ACC using HND under TTLT versus ED based ACC with the support of ARLs. It has been shown that the presented charting structure based on HND detects process variation more quickly than the chart based on ED. The usage of a planned chart based on HEPD is exhibited with the aid of simulated data and real-life examples that fully support the implementation of the proposed idea. The inclusion of multiple real-life examples in the study strengthens the research and improves the potential applicability of the proposed control chart in a broader range of scenarios.

The suggested chart can undoubtedly be stretched out to other life data distributions like Alpha power inverse Weibull distribution proposed by Ref.^30^, and the generalized odd Burr III family of distributions suggested by Ref.^31^. for further research. In conclusion, the proposed ACC using HND and HEPD based on TTLT is an effective method to detect process variation in manufacturing processes. The results of OOC ARLs and comparison with ED based ACC have shown the superiority of the proposed methods. The application of the proposed charting structure in real-life examples further confirms its practicality. The extension of the proposed method to other life data distributions opens up new avenues for future research.

## Data Availability

The data is given in the paper.
